# Altered DNA methylation in liver and adipose tissues derived from individuals with obesity and type 2 diabetes

**DOI:** 10.1186/s12881-018-0542-8

**Published:** 2018-02-21

**Authors:** Francisco Barajas-Olmos, Federico Centeno-Cruz, Carlos Zerrweck, Iván Imaz-Rosshandler, Angélica Martínez-Hernández, Emilio J. Cordova, Claudia Rangel-Escareño, Faustino Gálvez, Armando Castillo, Hernán Maydón, Francisco Campos, Diana Gabriela Maldonado-Pintado, Lorena Orozco

**Affiliations:** 1 Inmunogenomics and Metabolic Disease Laboratory, Instituto Nacional de Medicina Genómica, SS México City, Mexico; 2 Clínica Integral de Cirugía para la Obesidad y Enfermedades Metabólicas, Hospital General Tláhuac, Secretaría de Salud de la CDMX, México City, Mexico; 3 Computational Genomics Consortium, Instituto Nacional de Medicina Genómica, SS México City, Mexico; 4 Hospital Ángeles Pedregal, México City, Mexico; 5 Clínica Integral de Cirugía para la Obesidad y Enfermedades Metabólicas, Hospital General Dr. Rubén Leñero, Secretaría de Salud de la CDMX, México City, Mexico

**Keywords:** Type 2 diabetes, DNA methylation, Gene expression, Adipose tissue, and liver tissue

## Abstract

**Background:**

Obesity is a well-recognized risk factor for insulin resistance and type 2 diabetes (T2D), although the precise mechanisms underlying the relationship remain unknown. In this study we identified alterations of DNA methylation influencing T2D pathogenesis, in subcutaneous and visceral adipose tissues, liver, and blood from individuals with obesity.

**Methods:**

The study included individuals with obesity, with and without T2D. From these patients, we obtained samples of liver tissue (*n* = 16), visceral and subcutaneous adipose tissues (*n* = 30), and peripheral blood (*n* = 38). We analyzed DNA methylation using Illumina Infinium Human Methylation arrays, and gene expression profiles using HumanHT-12 Expression BeadChip Arrays.

**Results:**

Analysis of DNA methylation profiles revealed several loci with differential methylation between individuals with and without T2D, in all tissues. Aberrant DNA methylation was mainly found in the liver and visceral adipose tissue. Gene ontology analysis of genes with altered DNA methylation revealed enriched terms related to glucose metabolism, lipid metabolism, cell cycle regulation, and response to wounding. An inverse correlation between altered methylation and gene expression in the four tissues was found in a subset of genes, which were related to insulin resistance, adipogenesis, fat storage, and inflammation.

**Conclusions:**

Our present findings provide additional evidence that aberrant DNA methylation may be a relevant mechanism involved in T2D pathogenesis among individuals with obesity.

**Electronic supplementary material:**

The online version of this article (10.1186/s12881-018-0542-8) contains supplementary material, which is available to authorized users.

## Background

Recent decades have seen an accelerated increase of the worldwide prevalence of overweight and obesity [1]. At this time, the leading causes of adult mortality are co-morbid conditions associated with obesity, such as cardiovascular diseases, T2D, and cancer [2]. Excessive fat accumulation induces metabolic derangements, of which insulin resistance is the most prominent, often leading to T2D [3]. The close link between obesity and altered insulin signaling is mediated by several mechanisms, including ectopic lipid accumulation, low-grade inflammation, and altered adipokine production [4]. Involvement of insulin resistance in the progression to diabetes is specific in each tissue. In the adipocyte, the production of fatty acids increases, which together with the rise of circulating fatty acids, hepatic glucose release and activation of the immune system synergize the deterioration of pancreatic beta cells [4, 5]. The high levels of circulating fatty acids can result in insulin resistance in skeletal muscle and liver.

However, although epidemiological and pathophysiological studies demonstrate that T2D and obesity are highly interrelated, the severity of insulin resistance varies considerably among individuals with obesity, in fact many of them do not progress to the diabetic state [6]. Both obesity and T2D are associated with many polymorphisms, but they share only a few susceptibility loci [7]. Despite enormous progress, we still lack sufficient understanding of the precise molecular mechanisms underlying T2D pathophysiology, particularly regarding mechanisms involving metabolic pathways in insulin target tissues [8].

Recent evidence suggests that tissue-specific transcriptional gene regulation by epigenetic factors plays a role in the development of insulin resistance and diabetes among individuals with obesity [9–12]. DNA methylation, one of the key epigenetic factors, is involved in the modulation of gene expression, mainly through the modulation of DNA-protein interactions [13, 14]. DNA methylation could provide a link between environmental influences, genetic factors and the development of defects in insulin signaling [15]. Increasing evidence in several populations, have assessed DNA methylation alterations in various tissues of T2D patients. It has been found altered methylation in genes related to beta cell survival in pancreas [16], in several TD2 related genes in subcutaneous adipose tissue [17, 18] and in genes related to glycolytic and lipogenic pathways in liver tissue [19]. To our knowledge, there are few reports of methylation alterations in visceral adipose tissue, although in insulin resistant obese patients the findings highlight genes related to immune response and cell adhesion process [20]. Here, we reported evidences for altered DNA methylation in visceral and subcutaneous adipose tissues, liver and blood from individuals with both obesity and T2D and the effects of these alterations in gene expression patterns.

## Methods

### Subjects and samples

Patients with a body mass index (BMI) of ≥35 kg/m^2^ were recruited from the Surgery Integral Clinics for Obesity and Metabolic Diseases at Tláhuac, Ruben Leñero and Ángeles del Pedregal Hospitals in Mexico City. All the patients were clinically evaluated before and during the surgery and no patients with important liver diseases were detected (such as NASH, fibrosis or cirrhosis). If any evidences of macroscopic pathological findings were found, no sample was taken for the study. Participants were classified as controls (non-diabetic individuals with obesity, NDO) or as cases (diabetic individuals with obesity, DO). All T2D patients fulfilled the American Diabetes Association criteria for diagnosis [21]. None of the patients received insulin therapy, although 15 of 23 DO and 5 of 23 NDO were under metformin treatment. Individuals with substance abuse (alcohol, tobacco or drugs) were also excluded from the study.

Cases and controls were matched according to gender, age, and BMI. Table 1 showed the characteristics of the patients at the time of surgery. All tissues were obtained during bariatric surgery procedures. Tissues were obtained from a group of 46 participants of which 23 were DO and 23 NDO. From these patients we obtained 30 visceral and subcutaneous adipose tissue samples and 16 liver biopsies. Blood samples were drawn from 38 patients. The samples were preserved in RNAlater (SIGMA-ALDRICH, St. Louis MO, USA) at − 70 °C until nucleic acid extraction.

**Table 1:** Anthropometric data of non-diabetic individuals with obesity and diabetic individuals with obesity

Data	Non-diabetic individuals with obesity	Diabetic individuals with obesity	p-value
n (woman/man)	16/7	16/7	–
Age	40.96 ± 6.19	41.75 ± 9.93	0.71
BMI	42.95 ± 6.06	41.19 ± 5.51	0.39
DBP	75.6 ± 7.28	72.67 ± 9.38	0.48
SBP	124.7 ± 15.11	120.33 ± 15.63	0.68
Total cholesterol	170.11 ± 33.73	205.11 ± 51.58	0.18
Triglycerides	148.28 ± 62.6	193.44 ± 98.61	0.14
HDL-cholesterol	33.37 ± 11.28	37.03 ± 11.37	0.33
Fasting glucose	84.35 ± 8.68	123.1 ± 28.52	2.63 × 10^−07^
%HB1Ac	5.35 ± 0.19	6.93 ± 0.94	1.03 × 10^−06^
INS	12.99 ± 6.59	18.23 ± 19.01	0.38
HOMA %B	155.92 ± 61.21	83.06 ± 39.54	2.37 × 10^−05^

Data are expressed as average value ± SD. Age in years; BMI: body mass index (kg/m^2^); Fasting glucose, Total cholesterol, HDL-cholesterol and Triglycerides, in mg/dl; INS: insulin, μU/ml; DBP: diastolic blood pressure in mmHg; SBP: systolic blood pressure in mmHg; HOMA: homeostatic model assessment, beta cell function (%B), Statistical analysis was performed by Mann-Whitney U test

### DNA extraction and quality control

DNA was extracted from whole blood samples using the GentraPuregene Blood Kit (Qiagen, Valencia CA, USA) and from solid tissues using the QIAamp DNA Mini Kit (Qiagen, Valencia CA, USA). DNA extractions were performed following the manufacturer’s instructions.

### RNA extraction

Total RNA was extracted from whole blood and liver tissue using Trizol reagent (Life Technologies, Inc., Rockville, MD, USA). RNA was extracted from adipose tissues using the RNeasy Lipid Tissue Mini Kit (Qiagen, Valencia CA, USA). RNA extractions were performed following the manufacturer’s instructions. RNA integrity was assayed using the Agilent 2100 Bioanalyzer system, following the manufacturer’s instructions. Only samples with an RNA integrity number (RIN) above 8 were analyzed using microarrays.

### Genome-wide methylation assay

We used the HumanMethylation27 BeadChip (Illumina, Inc., San Diego, CA, USA), which covered 27,578 CpG sites (CpGs) around 14,495 genes throughout the genome. Cases and controls were randomly located among the BeadChips. Arrays were processed following the Illumina Infinium Methylation assay protocol, and were scanned in an Illumina iScan. The array results were visualized using Illumina GenomeStudio software (V2011.1). We only included samples which passed quality control (QC) of internal control probes. CpGs in sex chromosomes were excluded, and the subsequent quality-filtered analyses included 26,486 CpGs. The methylation value was calculated as Beta, ranging from 0 (unmethylated) to 1 (completely methylated). The Beta value represents the ratio of the methylated probe intensity and the sum of methylated and unmethylated probe intensities.

### Genome-wide gene expression analysis

We amplified the extracted RNA (750 ng) into biotinylated cRNA using the Illumina TotalPrep RNA Amplification Kit (Life Technologies, Foster City, CA, USA). Samples were analyzed using Illumina HumanHT-12 v4 Expression BeadChips (Illumina, Inc., San Diego, CA, USA), which target more than 47,000 probes. Arrays were processed following the standard Illumina protocol. We used GenomeStudio software to verify quality standards for hybridization, labeling, staining, background signal, and basal level of housekeeping gene expression for each chip.

### Statistical analysis

Computation and statistical analyses were performed using the R, version 3.1 (http://www.r-project.org). The Lumi package was used to normalize the raw intensities of DNA methylation and gene expression arrays (robust spline normalization) [22]. Then, Beta as well as log_2_ transformed gene expression values were computed and Linear Models for Microarray Data (Limma) from Bioconductor were implemented in order to compare between DO and NDO phenotypes [23]. A differentially methylated CpG site (DMCs) was defined as one that reached at least a 5% of difference (Delta-Beta) in absolute mean methylation, with an unadjusted *p* value of < 0.05. The cell-type heterogeneity adjustment for blood sample was performed using the ChAMP package, this include RefbaseEWAS method that uses a reference-database of DNA methylation from whole blood to detect cell proportion and correct the cell heterogeneity on the data [24]. Expression probes with a |log fold-change| of > 0.8 and *p* < 0.05, were considered to show altered gene expression. To assess the correlation between alteration of DNA methylation and differential gene expression, we followed the strategy previously published by Noushmehr (2010) [25], which involves assigning the direction of the change (positive or negative) from delta Beta or log fold-change to the significance of change (*p* value), and then graphing it as log_10_ was followed. We focused on probes that were significantly down-regulated and significantly hypermethylated, and on probes that were significantly up-regulated and significantly hypomethylated.

### Pathway analyses

To obtain enriched Gene Ontology (Biological Process), were used all gene lists from Additional file 1: Tables S1-S4, Tables S8-S11, and Tables S12-S15. Pathway analysis was performed using the Database for Annotation, Visualization, and Integrated Discovery (DAVID 6.8), only terms with adjusted *p*-value < 0.05 are listed [26].

## Results

### Samples

Samples were obtained from the NDO (*n* = 23) and DO (*n* = 23) individuals, gender-, age-, and BMI-matched. As shown in Table 1, fasting glucose, %HbA1c, and HOMA-B were altered among DO patients. DO shown higher average values of total cholesterol and triglycerides, although these did not significantly differ between both groups.

### Global DNA methylation

We analyzed Global DNA methylation in liver tissue (LT), visceral adipose tissue (VAT), subcutaneous adipose tissue (SAT) and whole blood (WB). Global methylation patterns were highly correlated with other samples of the same tissue type from different individuals, independent of their T2D status. In fact, DNA methylation patterns were highly conserved among all analyzed tissue samples (Additional file 1: Figure S1). Comparison of the average methylation levels among tissue types revealed the highest correlation between SAT and VAT samples (0.99) and the lowest correlation between LT and WB samples (0.94) (Additional file 1: Figure S2).

### Differential DNA methylation between DO and NDO patients

**Fig. 1:**
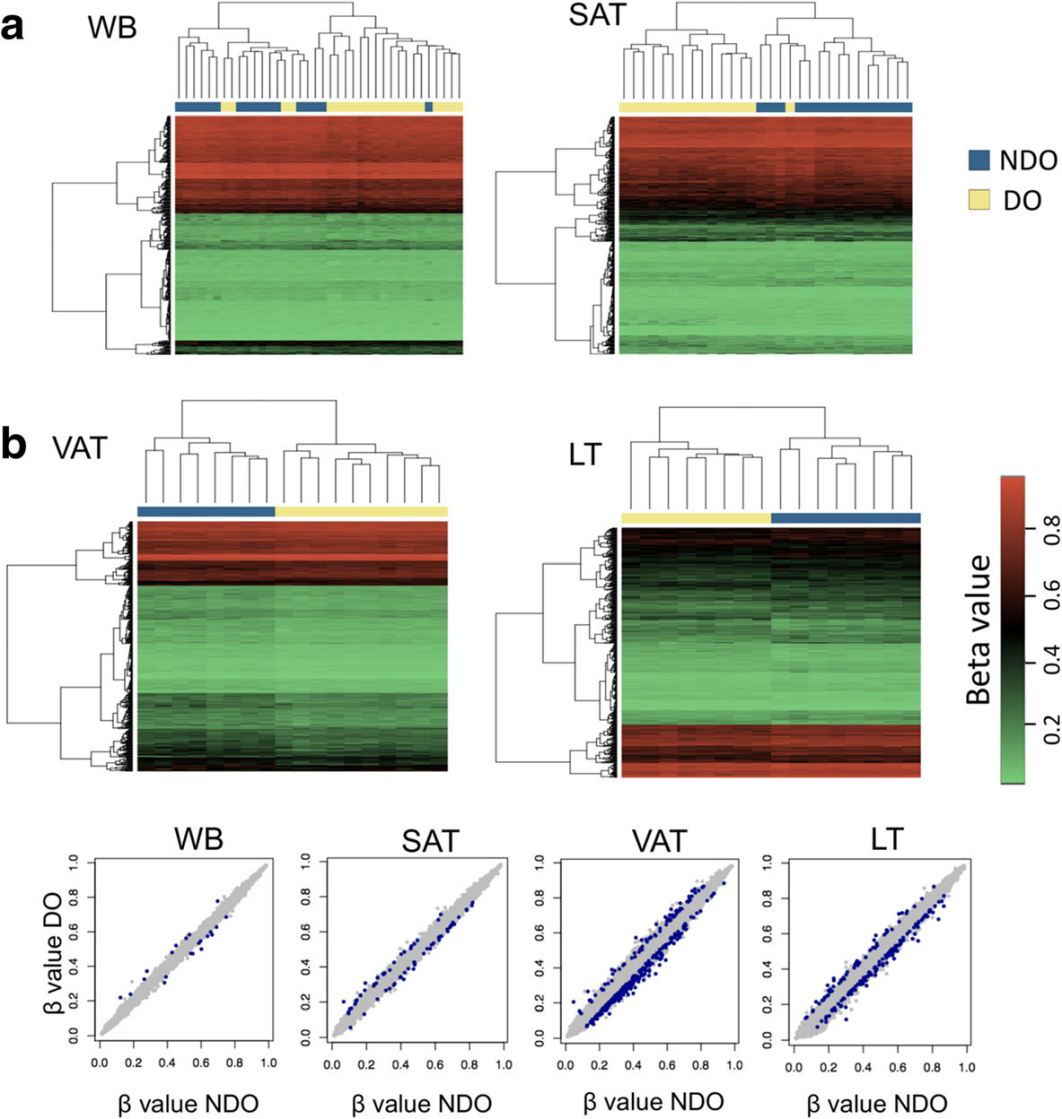
Differential methylation profiles from individuals with obesity and T2D. (**a**) Heat map shows the top 500 differentially methylated CpG sites. Hierarchical clustering distinguishes NDO (blue bar) from DO samples (yellow bar). (**b**) Scatter plot of DNA methylation shows the average beta values of NDO and DO patients. DMCs are highlighted in blue

WB, SAT, VAT, and LT samples showed differential DNA methylation profiles between DO and NDO individuals. We identified two main clusters in the four analyzed tissues, by applying hierarchical clustering analysis based on the top 500 differentially methylated CpG sites between DO and NDO, regardless of the value of delta beta. However, both groups were fully discriminated only in VAT and LT analysis. In SAT analysis, one DO individual was included with the NDO subjects; and in WB analysis, several DO individuals were included in the NDO control group (Fig. 1a).

We selected the DMCs in each tissue for deeper analysis. We observed the highest amount of DMCs in VAT (340 DMCs, including 78 positive and 262 negative) and LT (185 DMCs, including 57 positive, and 128 negative), followed by SAT (68 DMCs, including 29 positive and 39 negative) and WB (21 DMCs, including 9 positive and 12 negative) (Fig. 1b). Several of the observed DMCs were located in genes related to T2D or obesity, such as *ALOX12*, *PAMR1*, and *GNAS* in WB; *IRS1*, *LEP*, and *ADIPOQ* in SAT; *LCAT*, *FOXA2*, *KCNQ1*, and *GCKR* in VAT; and *PKD4*, *HNF4A*, *XBP1*, and *PON1* in LT.

Interestingly, comparison between the DO and NDO groups showed that several DMCs were shared among tissues. DMCs located in *CCDC185*, *MTHFD2*, and *SUMF1* overlapped between SAT and VAT; DMCs located in *FUCA1*, *C4orf33*, *PRAP1*, *SNX4*, *TMEM109*, and *ZNF597* overlapped between VAT and LT; and DMCs in *MIA2* overlapped between SAT and LT. Moreover, WB also showed the DMCs in *PSMD5*, *PAMR1*, and *SUMO3* found in SAT, DMCs in *SLC9A2* found in VAT; and DMCs in *ALOX12* found in LT. DMCs in *PSMD5*, *SLC9A2*, and *ALOX12* showed a significant correlation between WB and the other tissues (Additional file 1: Figure S3). Additional file 1: Tables S1-S4, show the complete list of DMCs.

We performed gene ontology enrichment analysis using the genes with DMCs. The identified significant gene ontology terms in SAT were mainly related to lipid and carbohydrate metabolism and insulin response; those in VAT were mainly related to cell death, apoptosis, and cell cycle; and those in LT were mainly related to wound healing, coagulation, and cell growth (Additional file 1: Tables S5-S7). We found no significantly enriched gene ontology terms in WB.

### Effects of altered DNA methylation on gene expression

**Fig. 2:**
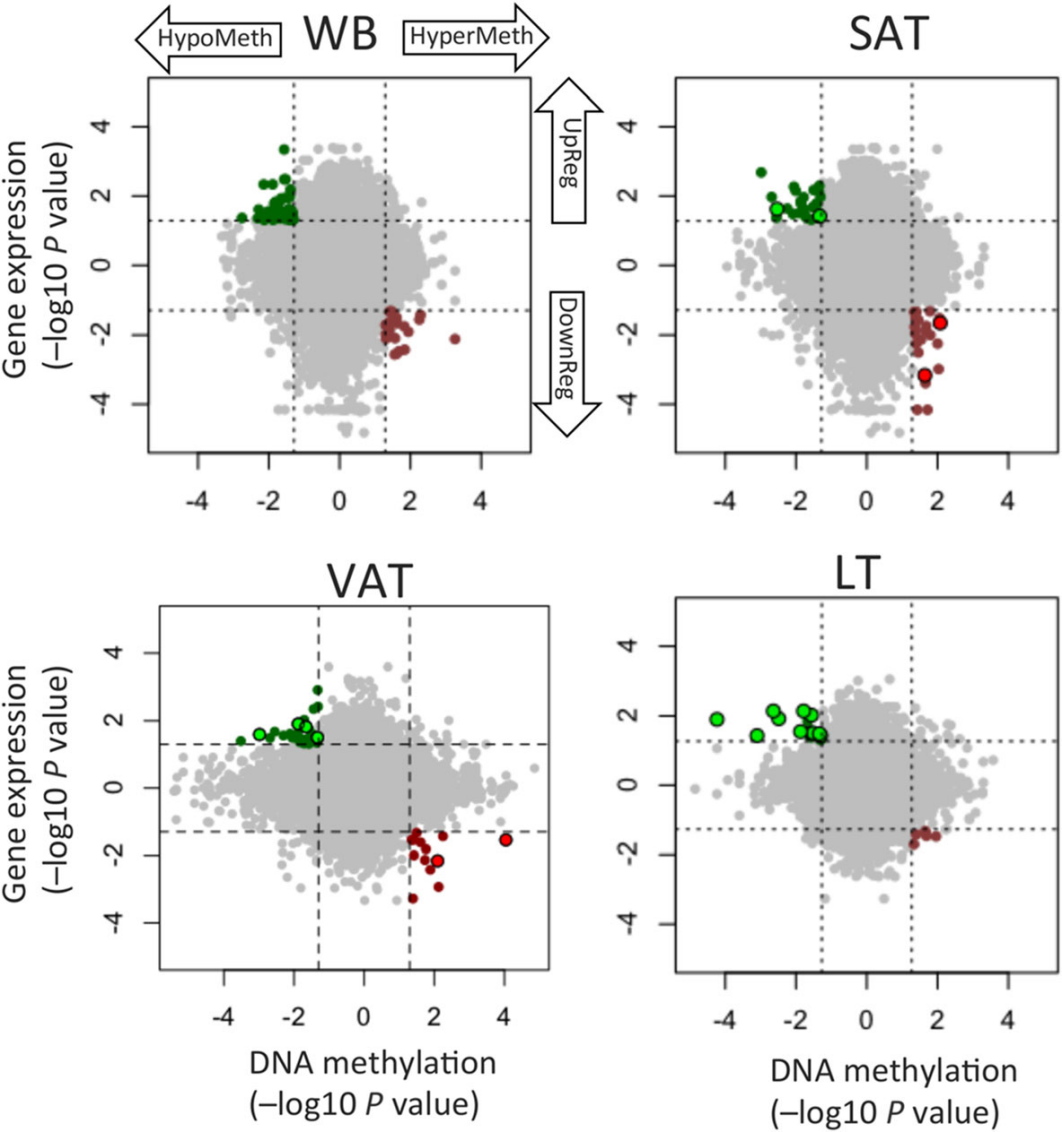
Starburst plot for the comparison of DNA methylation and gene expression. Dashed black lines indicate *p* value of 0.05. Red points indicate genes that are significantly down-regulated (DownReg) and hypermethylated (HyperMeth). Green points indicate genes that are significantly up-regulated (UpReg) and hypomethylated (HypoMeth). DMCs are highlighted with black contour

To understand the biological effects of altered methylation, we analyzed gene expression using HumanHT-12 v4 Expression BeadChips. Comparison between the DO and NDO groups revealed subtle differential gene expression in all analyzed tissues (Additional file 1: Tables S8-S11 and Figure S4). To determine whether the DNA methylation differences between DO and NDO led to functional effects in transcription, we investigated the correlation of DNA methylation with gene expression data, using a previously reported strategy [15]. Starburst plots in Fig. 2 show the genes that displayed an inverse correlation between altered DNA methylation and altered expression. Additional file 1: Tables S12-S15 show the complete list of these genes and the Additional file 1: Table S16 shows the GO enrichment analysis. Noteworthy, genes with the highest altered DNA methylation (>|5%|) and altered expression included *DST*, *MGAT4C*, *LEP* and *ZNF3* in SAT; *BRDT*, *C14orf105*, *EDNRB*, *HMP19*, *PSG6* and *SNX4* in VAT; and *SYT7*, *LTBR*, *CATSPER2*, *LPAL2*, *NCALD*, *ZDHHC11*, *LGTN*, *OXT* and *PRSS21* in LT. Most of these genes have not been previously related with T2D.

## Discussion

Multiple studies have highlighted the importance of genetic factors in the etiology of metabolic diseases; however, only a small fraction of susceptibility genes are identified and few of them are reportedly shared among these diseases. Emerging evidence supports the role of epigenetic mechanisms as a crucial interface between genetic and environmental influences [11]. Some studies suggest that altered DNA methylation may be critical in the development of obesity and T2D [12, 27, 28–30]. However, tissue specific mechanisms involved in obesity and T2D are still unclear. To deepen our understanding of the epigenomic mechanisms underlying T2D development, here we analyzed the methylation and transcription profiles in blood, liver, and subcutaneous and visceral adipose tissue samples from individuals with obesity, with and without T2D.

Comparison of DNA methylation profiles revealed a high correlation among tissue of the same type (Additional file 1: Figure S1), independent of T2D status, showing an epigenetic footprint for each tissue type, supporting the evidence that the developmental origin and tissue environment are the primary determinants of global DNA methylation patterns [31]. According whit this conception, our comparison of DNA methylation profiles among different tissues showed the strongest correlation between subcutaneous and visceral adipose tissues (Additional file 1: Figure S2), reflecting the high functional similarity between them. A high correlation between genome wide-DNA methylation of these tissues was previously reported [32]. When we stratified by diabetic status, DNA methylation profiles revealed significant differences in all analyzed tissues. Adjustment for cell-type heterogeneity was performed only in blood sample, since most of the broadly applied methods to correct for cellular heterogeneity are based on methylation datasets from blood. A limitation is the lack of a reliable method for adjusting the variation in cellular composition in fat and liver tissues. Another limitation could be the relatively reduced number of sample which could impact in the statistical power for identification of altered methylation sites associated to T2D.

In agreement with previous findings, the most of the altered DNA methylation sites showed hypomethylation [16, 18–20]. Altered DNA methylation was mainly observed in LT and in VAT, which enable clustering by T2D status, as previously described [19, 20]. The lack of available data for the accurate diagnosis and staging of NAFLD/NASH involves a risk of stratification within the groups, although in this study we do not identified CpGs reported as altered in the progression of NAFLD/NASH [33, 34]. SAT also showed differential methylation between the DO and NDO groups, although one of DO cases was included among the controls, otherwise, methylation analysis of WB did not cluster patients according to T2D status. These findings, highlight the relevance of insulin target tissues in T2D physiopathology and contributing to the evidence of the importance of the epigenetic role in the pathophysiology of these diseases [16].

As expected, several of the identified DMCs involved genes previously reported with altered methylation profiles in T2D such as *ADIPOQ*, *IRS1*, and *LEP* in SAT and *KCNQ1* in VAT [35]. Remarkably, we found DMCs located in the genes *LCAT and FOXA2 in VAT,* and *PON1 and FGF21* in LT*,* which have been genetically associated with metabolic traits, however, there are not enough evidences of their altered methylation in these entities [36–38]. Some of these genes have been involved in lipid metabolism pathways, and although the DO showed higher cholesterol and triglycerides serum levels, there were not significant differences between both groups, hence it is possible that their epigenetic regulation could be involved in the interplay between the alteration of lipid metabolism and the development of T2D [39, 40].

Although in this study the majority of DMCs were tissue specific, some of them were shared among tissues, perhaps representing common pathway in insulin-target tissues and strengthening the previous suggestion that adverse factors may similarly impact DNA methylation in different tissues [12]. Interestingly, the altered methylation of *ALOX12* (LT), *PSMD5* (SAT), and *SLC9A2* (VAT) was significantly correlated between internal tissues and blood, suggesting that particular DMCs could be useful biomarkers. Further studies are needed to delimitate the regions and CpG sites in blood that mirror DNA methylation in other tissues. In this study 15/23 DO patients and 5/23 NDO patients were under metformin treatment. The effect of metformin on DNA methylation is still unclear and as far as we know, the unique report on DNA methylation in diabetic patients treated with metformin showed hypomethylation of *SLC22A1*, *SLC22A3* and *SLC47A1* in liver, however, we found none of these genes altered in our patients [41].

On the other hand, tissue-specific alterations in gene expression have been reported in T2D [42–44], although there are few reports involving the epigenomic regulation on gene expression. Interestingly, all tissues of T2D patients showed altered expression of genes involved in the inflammation response—such as *SOCS1* in WB; *CCL20* and *SLAMF1* in SAT; *ACP5*, *FOS*, and *JUN* in VAT; and *PLA2G2A* in LT, showing the relevance of the systemic chronic inflammation in obesity and T2D [45]. We also found altered expression of genes involved in adipogenesis (*AMFR, ACP5*, and *FOSB*) [46–48], a process that is also widely reported in both diseases [49].

For better understanding of the role of epigenetic dysregulation in T2D, we integrated the methylation and expression data. We found a direct impact of the altered DNA methylation on expression of a subset of genes. The pathways enriched with these genes included several involved in T2D pathogenesis, such as adipogenesis, regulation of apoptosis, mitochondrion organization, oxygen transport, immune response and insulin resistance. In addition, we found DNA methylation and expression altered in genes having important roles in fat storage and that had not previously been reported to show epigenetic alteration in TD2—including *SCT* in WB and SAT, *OPRM1* in SAT, *IRX3* in VAT, and *NRG4* in LT. Our findings are in line with previous reports that have pointed to epigenetic altered glycolytic and lipogenic pathways in T2D patients [19].

## Conclusions

In summary, here we provide a catalog of genes affected by altered methylation in liver, visceral and adipose tissues, and peripheral blood samples from patients with obesity and T2D. Our present results demonstrate tissue-specific epigenetic alterations in individuals with obesity and T2D, and highlight the roles of liver and adipose tissue in metabolic disease development. Altered DNA methylation may directly impact transcription, consequently altering several pathways related to metabolic processes involved in T2D etiopathogenesis. Here we present new evidence that altered DNA methylation is a mechanism that affects these processes in individuals with obesity developing T2D. Overall, the available data suggest that altered methylation of genes involved in cell metabolism could be a crucial part of the mechanisms behind the comorbidity of metabolic diseases. Further studies are needed to integrate the available genetic and epigenetic data to improve our understanding of the intriguing interplay between genetic and environmental mechanisms in relation to the development of complex traits, like T2D.

## Additional file

Additional file 1 **Figure S1.** Clustering of methylation data from tissue samples from individuals with obesity. **Figure S2.** Comparison of methylation averages among tissue types. **Figure S3.** Comparison of DMCs between different tissues. **Figure S4.** Differential gene expression. **Table S1.** List of DMCs in WB in the comparison between the DO and NDO groups. **Table S2.** List of DMCs in SAT in the comparison between the DO and NDO groups. **Table S3.** List of DMCs in VAT in the comparison between the DO and NDO groups. **Table S4.** List of DMCs in LT in the comparison between the DO and NDO groups. **Table S5.** Gene ontology enrichment analysis using the genes with DMCs in SAT. **Table S6.** Gene ontology enrichment analysis using the genes with DMCs in VAT. **Table S7.** Gene ontology enrichment analysis using the genes with DMCs in LT. **Table S8.** Differential gene expression in WB in the comparison between DO and NDO groups. **Table S9.** Differential gene expression in SAT in the comparison between DO and NDO groups. **Table S10.** Differential gene expression in VAT in the comparison between DO and NDO groups. **Table S11.** Differential gene expression in LT in the comparison between DO and NDO groups. **Table S12.** List of genes with correlation between alteration of DNA methylation and differential gene expression in WB. **Table S13.** List of genes with correlation between alteration of DNA methylation and differential gene expression in SAT. **Table S14.** List of genes with correlation between alteration of DNA methylation and differential gene expression in VAT. **Table S15.** List of genes with correlation between alteration of DNA methylation and differential gene expression in LT. **Table S16.** Gene ontology enrichment analysis using the genes with correlation between alteration of DNA methylation and differential gene expression. (PDF 3440 kb)

## Abbreviations

CpGs: CpG sites
DMCs: Differentially methylated CpG sites
DO: Diabetic individuals with obesity
GO: Gene Ontology
LT: Liver tissue
NDO: non-diabetic individuals with obesity
SAT: Subcutaneous adipose tissue
T2D: Type 2 diabetes
VAT: Visceral adipose tissue
WB: Whole blood
