# Supplementary material for: Altered DNA methylation in liver and adipose tissues derived from individuals with obesity and type 2 diabetes
Source: BMC Med Genet. 2018 Feb 21;19:28. doi: 10.1186/s12881-018-0542-8 (PMC5822594; doi:10.1186/s12881-018-0542-8)
Supplement: Supplementary file 1 — Figure S1. Clustering of methylation data from tissue samples from individuals with obesity. Figure S2. Comparison of methylation averages among tissue types. Figure S3. Comparison of DMCs between different tissues. Figure S4. Differential gene expression. Table S1. List of DMCs in WB in the comparison between the DO and NDO groups. Table S2. List of DMCs in SAT in the comparison between the DO and NDO groups. Table S3. List of DMCs in VAT in the comparison between the DO and NDO groups. Table S4. List of DMCs in LT in the comparison between the DO and NDO groups. Table S5. Gene ontology enrichment analysis using the genes with DMCs in SAT. Table S6. Gene ontology enrichment analysis using the genes with DMCs in VAT. Table S7. Gene ontology enrichment analysis using the genes with DMCs in LT. Table S8. Differential gene expression in WB in the comparison between DO and NDO groups. Table S9. Differential gene expression in SAT in the comparison between DO and NDO groups. Table S10. Differential gene expression in VAT in the comparison between DO and NDO groups. Table S11. Differential gene expression in LT in the comparison between DO and NDO groups. Table S12. List of genes with correlation between alteration of DNA methylation and differential gene expression in WB. Table S13. List of genes with correlation between alteration of DNA methylation and differential gene expression in SAT. Table S14. List of genes with correlation between alteration of DNA methylation and differential gene expression in VAT. Table S15. List of genes with correlation between alteration of DNA methylation and differential gene expression in LT. Table S16. Gene ontology enrichment analysis using the genes with correlation between alteration of DNA methylation and differential gene expression. (PDF 3440 kb) [file 12881_2018_542_MOESM1_ESM.pdf]

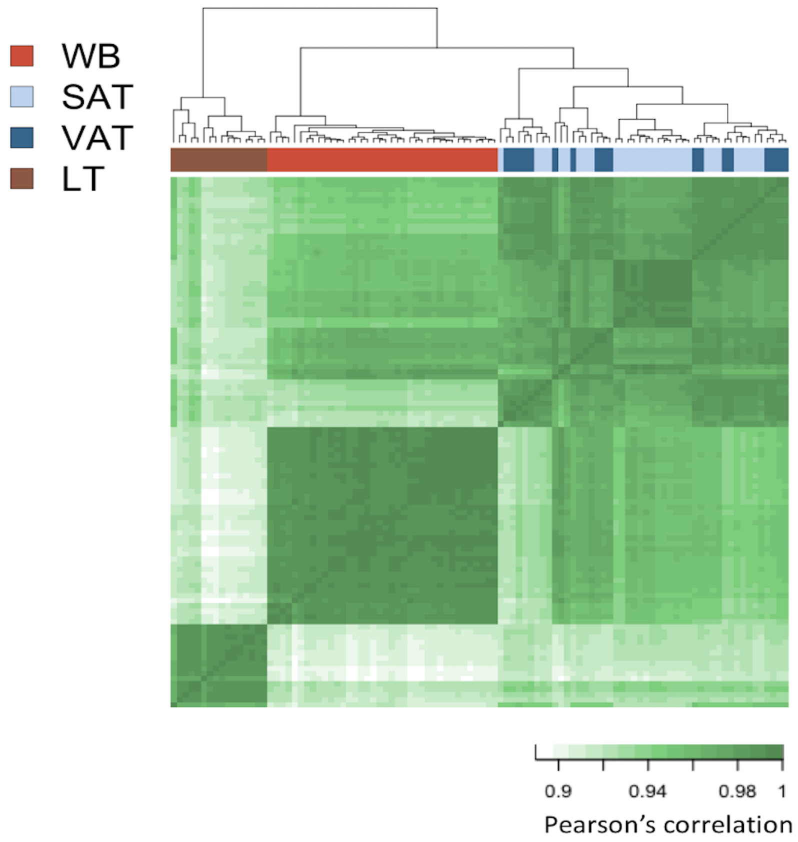

Figure S1. Clustering of methylation data from tissue samples from individuals with obesity. Pearson's correlation coefficient-based heat-map representation of methylation status of autosomal CpG sites among different tissues.

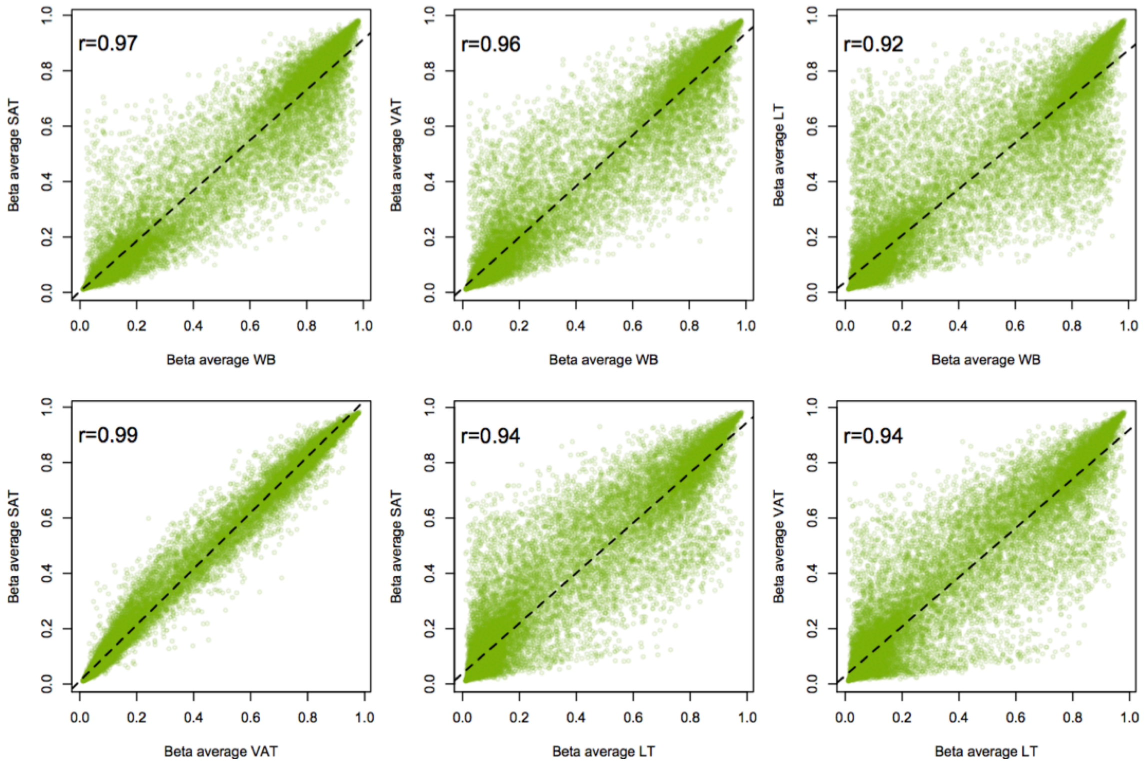

Figure S2. Comparison of methylation averages among tissue types. Scatter plots of comparison of DNA methylation levels between different tissue samples. Pearson's r values are denoted in the top left corner.

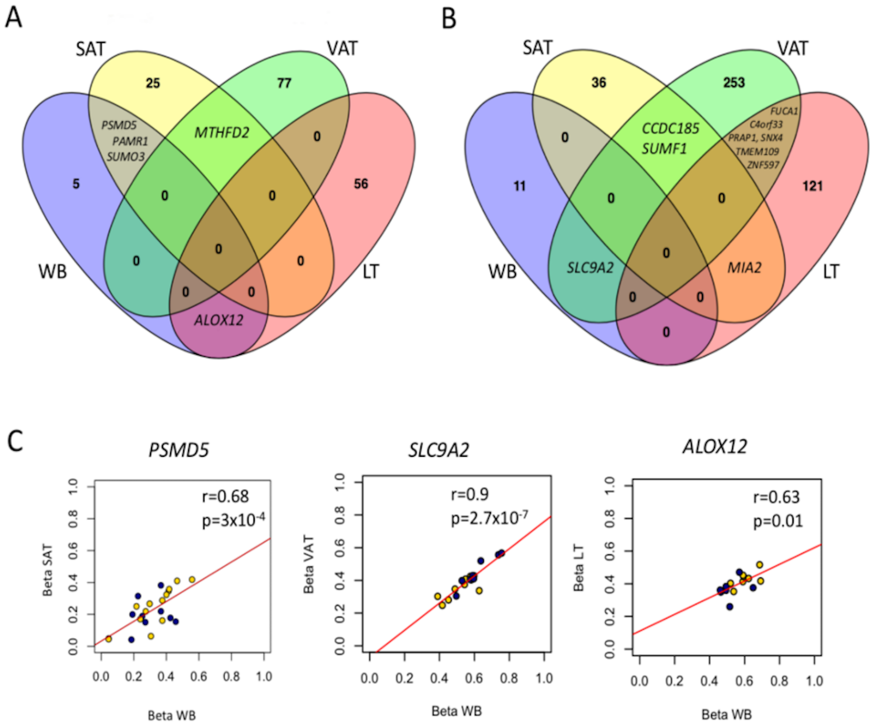

Figure S3. Comparison of DMCs between different tissues. (A) Venn diagram of positive DMCs. **(B)** Venn diagram of negative DMCs from WB. **(C)** Sites with correlation between WB and other tissues: *ALOX12 (*cg03760483), *PSMD5* (cg09419670), and *SLC9A2* (cg20050113).

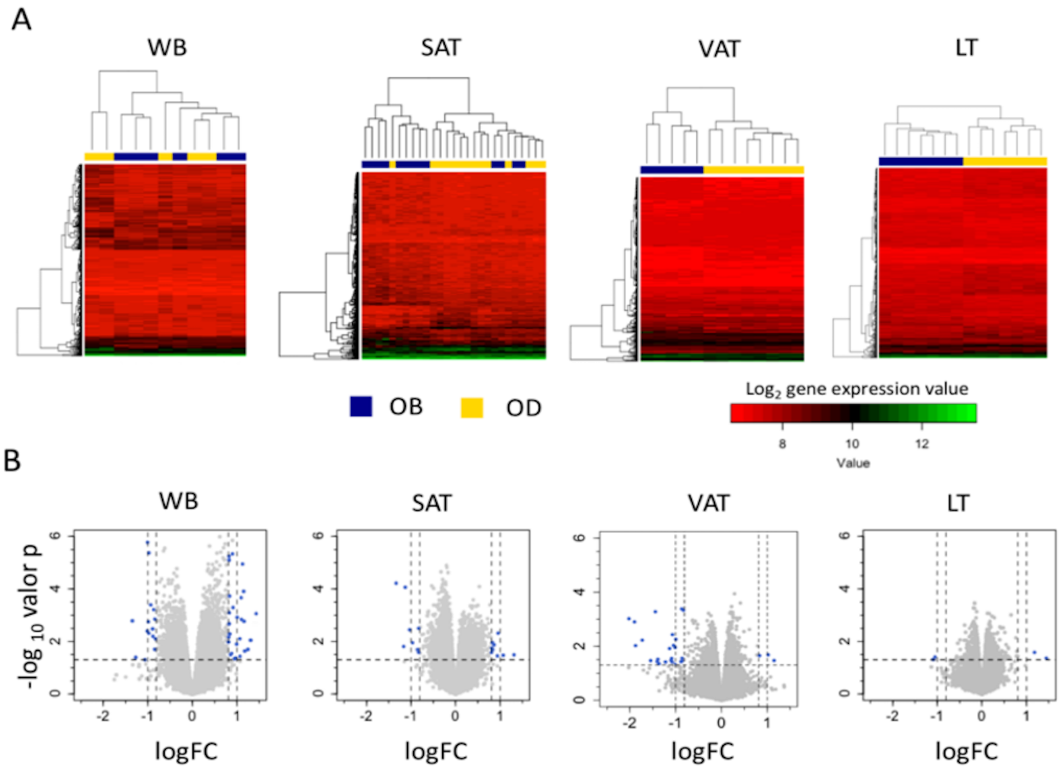

Figure S4. Differential gene expression. **(A)** Hierarchical clustering analysis of the top 500 identified probes that were differentially expressed between DO and NDO across tissues. The color bars at the top of the heat map indicate the patient status: yellow for DO, and blue for NDO. **(B)** Volcano plot of the log odds of differential expression vs. the log fold-change in gene expression between DO and NDO patients in WB, SAT, VAT, and LT. Probes with a |fold-change| of >0.8 and p<0.05 are highlighted in blue.

Table S1. List of DMCs in WB in the comparison between the DO and NDO groups

Target ID	Symbol	CHR	CPG Island	Beta DO	Beta OB	Delta Beta	*p*-value
cg13105904	*KHNYN*	14	TRUE	0.22	0.12	0.1	0.009
cg09419670	*PSMD5**	9	TRUE	0.37	0.28	0.09	0.008
cg21717724	*PSMD5*	9	TRUE	0.78	0.7	0.08	0.049
cg03760483	*ALOX12*	17	TRUE	0.52	0.45	0.07	0.003
cg21053323	*SUMO3*	21	TRUE	0.33	0.26	0.06	0.001
cg06873352	*STRADA*	17	TRUE	0.24	0.18	0.06	0.010
cg19728382	*STC2*	5	TRUE	0.58	0.53	0.05	0.009
cg19592945	*P2RXL1*	22	FALSE	0.48	0.43	0.05	0.033
cg14642338	*PAMR1*	11	FALSE	0.56	0.51	0.05	0.044
cg20050113	*SLC9A2*	2	TRUE	0.55	0.6	-0.05	0.048
cg06906435	*C14orf177*	14	FALSE	0.53	0.59	-0.05	0.039
cg02588309	*TTC33*	5	TRUE	0.34	0.39	-0.05	0.010
cg22686523	*FLJ25006*	17	FALSE	0.6	0.66	-0.06	0.048
cg13982505	*KCNJ13**	2	FALSE	0.69	0.75	-0.06	0.019
cg13033054	*LINC00846*	21	FALSE	0.57	0.64	-0.06	0.026
cg23439277	*PLCE1**	10	FALSE	0.63	0.69	-0.06	0.009
cg25853078	*OPCML**	11	FALSE	0.47	0.54	-0.06	0.003
cg13383491	*GPR85**	7	FALSE	0.47	0.55	-0.08	0.001
cg06771126	*HOP**	4	FALSE	0.3	0.38	-0.08	0.012
cg25997474	*ADH1C**	4	FALSE	0.58	0.66	-0.08	0.007
cg03606258	*GNAS*	20	TRUE	0.5	0.6	-0.1	0.038

*significant DMCs after adjusted by cell-type heterogeneity

Table S2. List of DMCs in SAT in the comparison between the DO and NDO groups

Target ID	Symbol	CHR	CPG Island	Beta DO	Beta OB	Delta Beta	*p*-value
cg17687883	*MTHFD2*	2	TRUE	0.2	0.07	0.14	0.011
cg09405083	*WBP1L*	10	FALSE	0.31	0.19	0.12	0.024
cg09419670	*PSMD5*	9	TRUE	0.27	0.19	0.08	0.024
cg14144305	*ALX4*	11	TRUE	0.34	0.26	0.08	0.007
cg04317399	*HOXA4*	7	TRUE	0.34	0.27	0.08	0.018
cg14642338	*PAMR1*	11	FALSE	0.47	0.4	0.07	0.006
cg00983520	*CPT1B*	22	TRUE	0.24	0.17	0.07	0.008
cg10190509	*CCL16*	17	FALSE	0.7	0.63	0.07	0.029
cg19393006	*TSC1*	9	FALSE	0.49	0.42	0.07	0.005
cg00186701	*TSPYL5*	8	TRUE	0.43	0.36	0.07	0.026

cg09025324	*SART2*	6	TRUE	0.2	0.14	0.06	0.03
cg08321346	*ANKMY1*	2	TRUE	0.21	0.14	0.06	0.034
cg25316898	*C18orf21*	18	TRUE	0.17	0.1	0.06	0.047
cg00648883	*EEF1DP3*	13	FALSE	0.36	0.3	0.06	0.028
cg17903316	*LOXHD1*	18	FALSE	0.36	0.3	0.06	0.047
cg26154999	*FAM124B*	2	TRUE	0.36	0.31	0.06	0.038
cg10098888	*IRS1*	2	TRUE	0.17	0.11	0.06	0.017
cg01193293	*SIGLEC7*	19	FALSE	0.52	0.46	0.06	0.05
cg22062068	*HNRNPG-T*	11	TRUE	0.55	0.5	0.06	0.043
cg12782180	*LEP*	7	TRUE	0.3	0.25	0.06	0.008
cg21053323	*SUMO3*	21	TRUE	0.19	0.13	0.06	0.042
cg00929606	*ZNF3*	7	TRUE	0.31	0.26	0.05	0.023
cg21019522	*SLC22A18*	11	FALSE	0.3	0.24	0.05	0.017
cg24664957	*VPS37B*	12	TRUE	0.51	0.46	0.05	0.003
cg11405695	*ATAD3C*	1	FALSE	0.53	0.48	0.05	0.028
cg06476606	*IFIT2*	10	FALSE	0.15	0.09	0.05	0.017
cg03160637	*DDB1*	11	TRUE	0.15	0.1	0.05	0.031
cg22222251	*WBSCR19*	7	FALSE	0.67	0.62	0.05	0.008
cg14679202	*L3MBTL2*	22	TRUE	0.39	0.34	0.05	0.027
cg03811411	*SGCD*	5	FALSE	0.65	0.7	-0.05	0.018
cg16504670	*DEF8*	16	TRUE	0.77	0.82	-0.05	0.009
cg05654163	*SLC39A2*	14	FALSE	0.62	0.67	-0.05	0.011
cg03190825	*CYP4F11*	19	FALSE	0.43	0.48	-0.05	0.023
cg21011830	*RPL18A*	19	TRUE	0.06	0.11	-0.05	0.011
cg04452713	*DST*	6	TRUE	0.26	0.31	-0.05	0.003
cg06563300	*SLC17A8*	12	FALSE	0.21	0.26	-0.05	0.018
cg07039113	*S100A9*	1	FALSE	0.73	0.78	-0.05	0.004
cg14290291	*MZT2B*	2	FALSE	0.43	0.48	-0.05	0.001
cg06112415	*CGREF1*	2	TRUE	0.37	0.42	-0.05	<0.001
cg24603941	*MIA2*	14	FALSE	0.68	0.74	-0.05	0.003
cg13980719	*TNP1*	2	FALSE	0.5	0.55	-0.05	0.022
cg24041453	*CCDC11*	18	TRUE	0.13	0.19	-0.05	0.024
cg01031251	*RPS6KA1*	1	TRUE	0.57	0.62	-0.05	0.004
cg05333568	*CCDC185*	1	TRUE	0.28	0.34	-0.05	0.043
cg04893119	*PI15*	8	FALSE	0.57	0.63	-0.05	0.031
cg03573747	*ADIPOQ*	3	FALSE	0.73	0.78	-0.06	0.006
cg00626466	*GNS*	12	TRUE	0.54	0.59	-0.06	0.022
cg05885720	*EMP1*	12	FALSE	0.63	0.69	-0.06	0.007
cg20781967	*NINJ2*	12	FALSE	0.34	0.4	-0.06	0.032

cg24833277	*FAM83A*	8	TRUE	0.64	0.7	-0.06	0.041
cg21048669	*CLEC3A*	16	FALSE	0.48	0.53	-0.06	0.026
cg27525902	*FGF7*	15	FALSE	0.52	0.58	-0.06	0.028
cg21550483	*CLEC10A*	17	FALSE	0.39	0.44	-0.06	0.043
cg19592945	*P2RXL1*	22	FALSE	0.34	0.4	-0.06	0.047
cg18847227	*SUMF1*	3	TRUE	0.42	0.48	-0.06	0.006
cg21686987	*CTRB1*	16	FALSE	0.75	0.81	-0.06	<0.001
cg03793778	*LGALS7*	19	FALSE	0.57	0.63	-0.06	0.044
cg18344063	*MGAT4C*	12	FALSE	0.57	0.64	-0.06	0.047
cg21832243	*TTC3*	21	FALSE	0.3	0.36	-0.06	0.038
cg00673191	*DOPEY2*	21	FALSE	0.3	0.36	-0.06	0.038
cg15302379	*KAZALD1*	10	TRUE	0.56	0.62	-0.07	0.001
cg21126707	*MYF5*	12	TRUE	0.5	0.57	-0.07	0.001
cg05293216	*FANCG*	9	TRUE	0.6	0.67	-0.07	0.01
cg26185508	*CDCP2*	1	TRUE	0.41	0.48	-0.07	0.025
cg07221454	*MS4A10*	11	FALSE	0.62	0.69	-0.07	0.041
cg15149938	*C22orf34*	22	FALSE	0.64	0.71	-0.07	0.007
cg02276665	*CTNNA1*	5	TRUE	0.39	0.47	-0.09	<0.001
cg02288165	*SN*	20	FALSE	0.33	0.42	-0.09	0.006

Table S3. List of DMCs in VAT in the comparison between the DO and NDO groups

Target ID	Symbol	CHR	CPG Island	Beta DO	Beta OB	Delta Beta	*p*-value
cg17687883	*MTHFD2*	2	TRUE	0.21	0.06	0.16	0.009
cg02964385	*STK38*	6	FALSE	0.58	0.42	0.16	0.036
cg15235832	*ACTR2*	2	TRUE	0.27	0.14	0.13	0.002
cg17872476	*VTI1A*	10	FALSE	0.40	0.28	0.12	0.001
cg22438810	*LCN2*	9	FALSE	0.66	0.54	0.11	0.023
cg01734338	*KCNQ1*	11	TRUE	0.74	0.63	0.11	0.001
cg04570669	*APIN*	4	FALSE	0.56	0.46	0.11	<0.001
cg11237738	*LINC01587*	4	FALSE	0.70	0.59	0.10	0.008
cg14074641	*ABCC12*	16	FALSE	0.39	0.30	0.09	0.003
cg01120761	*CLEC4C*	12	FALSE	0.77	0.68	0.09	0.040
cg02719634	*SLC22A18AS*	11	FALSE	0.84	0.75	0.09	0.032
cg20622019	*ADA*	20	TRUE	0.37	0.28	0.09	0.039
cg21755709	*PDXK*	21	TRUE	0.71	0.62	0.09	0.004
cg14732540	*BRDT*	1	TRUE	0.86	0.77	0.09	0.008
cg13906813	*HLA-DPA1*	6	FALSE	0.57	0.48	0.09	0.029
cg21781546	*ZNF142*	2	FALSE	0.70	0.63	0.08	<0.001
cg27212977	*DEFA6*	8	FALSE	0.81	0.73	0.08	<0.001
cg12218747	*SETD4*	21	TRUE	0.68	0.61	0.08	0.007
cg17994910	*CCDC30*	1	FALSE	0.49	0.41	0.08	0.013
cg17758148	*JMY*	5	FALSE	0.42	0.34	0.08	0.036
cg04601137	*ADAMTSL5*	19	FALSE	0.40	0.32	0.08	0.021
cg17496921	*TSPAN16*	19	FALSE	0.31	0.24	0.07	0.035
cg11376198	*AFAR3*	1	TRUE	0.22	0.14	0.07	0.030
cg21784940	*REPIN1*	7	FALSE	0.43	0.35	0.07	0.001
cg25112853	*HSC20*	22	FALSE	0.57	0.50	0.07	0.030
cg15792367	*KLK11*	19	FALSE	0.58	0.50	0.07	0.021
cg13525683	*TIAF1*	17	TRUE	0.73	0.66	0.07	0.010
cg13044136	*AES*	19	TRUE	0.16	0.09	0.07	0.011
cg11147886	*NFAT5*	16	FALSE	0.73	0.66	0.07	0.046
cg24612198	*CD3E*	11	FALSE	0.58	0.51	0.07	<0.001
cg18236297	*CYSLTR2*	13	FALSE	0.53	0.46	0.07	0.035
cg09325101	*OSTbeta*	15	FALSE	0.25	0.18	0.07	0.048
cg01817393	*GNAS*	20	TRUE	0.28	0.22	0.07	0.013
cg11584936	*BNIPL*	1	FALSE	0.67	0.60	0.07	0.045
cg08666623	*ST6GALNAC2*	17	FALSE	0.22	0.15	0.07	0.028
cg21644628	*HEATR3*	16	FALSE	0.63	0.56	0.07	0.009

cg19468534	*NUSAP1*	15	FALSE	0.55	0.49	0.07	<0.001
cg19103704	*FCGBP*	19	FALSE	0.46	0.39	0.07	0.024
cg27044702	*TPSAB1*	16	FALSE	0.70	0.64	0.07	0.042
cg00943909	*GNAS*	20	TRUE	0.51	0.44	0.07	0.029
cg04008843	*TTR*	18	FALSE	0.51	0.45	0.06	0.006
cg11151665	*PSG6*	19	FALSE	0.81	0.75	0.06	<0.001
cg19740969	*CXCL9*	4	FALSE	0.74	0.68	0.06	0.007
cg15862544	*EGFL4*	19	FALSE	0.83	0.77	0.06	0.021
cg23154064	*FAM208A*	3	FALSE	0.59	0.53	0.06	0.001
cg19279346	*LILRB2*	19	FALSE	0.68	0.62	0.06	0.005
cg09559551	*RSC1A1*	1	FALSE	0.78	0.72	0.06	0.024
cg02212280	*MMP1*	11	FALSE	0.80	0.74	0.06	<0.001
cg20812929	*DHRS4L2*	14	TRUE	0.31	0.25	0.06	0.022
cg08046471	*CXCL11*	4	FALSE	0.72	0.66	0.06	0.025
cg27016609	*STON1*	2	FALSE	0.49	0.44	0.06	0.001
cg26981881	*DEC1*	9	FALSE	0.73	0.67	0.06	0.039
cg09873510	*NLN*	5	FALSE	0.67	0.62	0.06	0.015
cg13982505	*KCNJ13*	2	FALSE	0.71	0.66	0.06	0.003
cg25803423	*C14orf119*	14	TRUE	0.15	0.09	0.06	0.028
cg27383744	*BRCA1*	17	TRUE	0.84	0.79	0.06	0.001
cg27661264	*GNAS*	20	TRUE	0.28	0.22	0.05	0.030
cg08972170	*Ells1*	7	TRUE	0.73	0.67	0.05	0.036
cg06776256	*GPRC5A*	12	TRUE	0.33	0.28	0.05	0.044
cg11003133	*AIM2*	1	FALSE	0.86	0.81	0.05	0.032
cg17606194	*SYCE1*	10	TRUE	0.47	0.41	0.05	0.037
cg14837082	*HRH1*	3	FALSE	0.65	0.60	0.05	0.015
cg11251498	*INDOL1*	8	FALSE	0.21	0.16	0.05	0.002
cg20895028	*CDH26*	20	FALSE	0.79	0.74	0.05	<0.001
cg16879115	*APOBEC1*	12	FALSE	0.70	0.65	0.05	0.021
cg25743584	*TIAF1*	17	FALSE	0.69	0.64	0.05	0.001
cg09964921	*KCNE1*	21	FALSE	0.78	0.73	0.05	0.002
cg22726338	*AKNAD1*	1	FALSE	0.68	0.62	0.05	0.011
cg27090087	*TINAG*	6	FALSE	0.51	0.45	0.05	0.032
cg17896229	*PROKR2*	20	TRUE	0.61	0.56	0.05	0.005
cg25372103	*DLL1*	6	TRUE	0.28	0.23	0.05	0.003
cg02131853	*TMEM156*	4	FALSE	0.70	0.65	0.05	<0.001
cg25420952	*AMBP*	9	FALSE	0.79	0.74	0.05	0.049
cg25650811	*CCDC129*	7	TRUE	0.59	0.54	0.05	0.008
cg20891917	*IFRD1*	7	FALSE	0.86	0.81	0.05	0.001

cg15344028	*ICOS*	2	FALSE	0.74	0.69	0.05	0.031
cg23580000	*ADCY7*	16	TRUE	0.87	0.82	0.05	0.040
cg00182461	*PPIL3*	2	TRUE	0.14	0.09	0.05	0.004
cg15674997	*GRM6*	5	TRUE	0.35	0.40	-0.05	0.001
cg10316764	*TAOK2*	16	TRUE	0.35	0.40	-0.05	0.038
cg12958778	*TBC1D14*	4	TRUE	0.47	0.52	-0.05	0.033
cg06637774	*P2RY6*	11	TRUE	0.24	0.29	-0.05	0.025
cg16601861	*ALDH1A1*	9	FALSE	0.34	0.39	-0.05	0.043
cg25509184	*CFTR*	7	TRUE	0.57	0.62	-0.05	0.024
cg00968931	*MRPL54*	19	FALSE	0.69	0.74	-0.05	0.025
cg17692403	*R3HDML*	20	FALSE	0.13	0.18	-0.05	0.006
cg00666746	*SYDE1*	19	TRUE	0.11	0.16	-0.05	0.001
cg10929387	*ITIH4*	3	FALSE	0.82	0.87	-0.05	0.017
cg09354267	*ATG5*	6	TRUE	0.28	0.34	-0.05	0.049
cg25938646	*SLITRK1*	13	TRUE	0.42	0.47	-0.05	0.023
cg00296685	*GTF2H4*	6	TRUE	0.44	0.49	-0.05	0.004
cg25356886	*CRYGD*	2	TRUE	0.42	0.47	-0.05	0.005
cg02337166	*NR1D1*	17	FALSE	0.10	0.15	-0.05	0.036
cg07880854	*FBLN7*	2	FALSE	0.17	0.22	-0.05	0.007
cg09893305	*HAPLN1*	5	FALSE	0.50	0.55	-0.05	0.040
cg27465566	*TRIO*	5	TRUE	0.30	0.35	-0.05	0.007
cg19903229	*C14orf105*	14	FALSE	0.57	0.62	-0.05	0.013
cg10537269	*TIMM9*	14	TRUE	0.20	0.25	-0.05	0.041
cg15343119	*GALR1*	18	TRUE	0.19	0.24	-0.05	0.004
cg08041019	*ATXN2*	12	TRUE	0.23	0.29	-0.05	0.005
cg10731149	*CYBRD1*	2	TRUE	0.59	0.64	-0.05	0.010
cg07657236	*KCNH8*	3	TRUE	0.18	0.24	-0.05	0.002
cg00567749	*CSPG2*	5	TRUE	0.34	0.40	-0.05	0.015
cg08445039	*FKBP9*	7	TRUE	0.13	0.18	-0.05	0.033
cg03464655	*SLC38A11*	2	TRUE	0.22	0.27	-0.05	0.026
cg10742801	*PRAP1*	10	FALSE	0.29	0.34	-0.05	0.007
cg02065387	*GABRA1*	5	FALSE	0.56	0.62	-0.05	0.047
cg22193702	*PADI1*	1	FALSE	0.82	0.87	-0.05	0.047
cg19188060	*PSMD2*	3	TRUE	0.13	0.19	-0.05	<0.001
cg09604428	*PB1*	3	FALSE	0.88	0.93	-0.05	0.025
cg02318535	*PPM1E*	17	TRUE	0.78	0.83	-0.05	0.048
cg09788239	*PCQAP*	22	TRUE	0.12	0.17	-0.05	0.003
cg03743584	*PRAP1*	10	FALSE	0.18	0.23	-0.05	0.020
cg08223748	*MEF2C*	5	TRUE	0.25	0.30	-0.05	0.008

cg07536847	*PAX7*	1	TRUE	0.16	0.21	-0.05	0.006
cg16468910	*SNX4*	3	TRUE	0.27	0.33	-0.05	0.022
cg16046951	*MKKS*	20	TRUE	0.21	0.26	-0.05	0.025
cg16168311	*APOA1BP*	1	TRUE	0.10	0.15	-0.05	0.001
cg16608652	*B3GALT2*	1	FALSE	0.81	0.86	-0.05	0.038
cg08261177	*ALDH16A1*	19	TRUE	0.07	0.12	-0.05	0.010
cg15014458	*LYPD3*	19	FALSE	0.24	0.30	-0.05	0.037
cg18411898	*RPRM*	2	TRUE	0.38	0.43	-0.05	0.002
cg17509872	*CNOT7*	8	FALSE	0.28	0.34	-0.05	0.018
cg15109571	*SUFU*	10	TRUE	0.07	0.13	-0.05	0.001
cg02727285	*RELB*	19	TRUE	0.38	0.43	-0.05	0.035
cg26815021	*SFRS2*	17	TRUE	0.17	0.23	-0.05	<0.001
cg08207256	*DNM2*	19	TRUE	0.17	0.22	-0.05	0.003
cg11885098	*EFNA2*	19	TRUE	0.23	0.28	-0.05	0.009
cg24958765	*RAB4B*	19	TRUE	0.15	0.20	-0.05	0.009
cg00021527	*TAF15*	17	TRUE	0.09	0.15	-0.05	0.010
cg19948393	*ANKRD33*	12	TRUE	0.48	0.54	-0.05	0.030
cg08054038	*PVRL2*	19	TRUE	0.46	0.51	-0.05	0.003
cg22430790	*FRMD6*	14	TRUE	0.20	0.25	-0.06	0.001
cg09209002	*SAS10*	4	TRUE	0.09	0.14	-0.06	0.027
cg18001427	*RWDD2B*	21	TRUE	0.16	0.22	-0.06	0.032
cg16195804	*C12orf65*	12	TRUE	0.16	0.21	-0.06	0.008
cg06150803	*TNFRSF19*	13	FALSE	0.23	0.28	-0.06	0.004
cg17166812	*NDUFS2*	1	FALSE	0.24	0.29	-0.06	0.041
cg23061257	*YIPF1*	1	TRUE	0.25	0.31	-0.06	0.019
cg06543018	*RBP1*	3	TRUE	0.19	0.25	-0.06	0.017
cg15350194	*FAM70B*	13	TRUE	0.28	0.34	-0.06	0.003
cg01190915	*MT2A*	16	TRUE	0.38	0.44	-0.06	0.036
cg02630888	*FBXL5*	4	TRUE	0.64	0.70	-0.06	0.034
cg20513206	*C19orf47*	19	TRUE	0.65	0.70	-0.06	0.048
cg12619509	*DNASE1L2*	16	TRUE	0.25	0.31	-0.06	<0.001
cg20867633	*GOLT1A*	1	TRUE	0.33	0.39	-0.06	0.002
cg20449692	*CLDN11*	3	TRUE	0.06	0.12	-0.06	0.002
cg23771929	*FREQ*	9	TRUE	0.19	0.25	-0.06	0.013
cg05154390	*MRPS15*	1	TRUE	0.17	0.23	-0.06	0.014
cg01333131	*CDC42BPB*	14	TRUE	0.13	0.19	-0.06	0.010
cg09164559	*KIAA1279*	10	TRUE	0.24	0.30	-0.06	0.047
cg08361238	*PTGFRN*	1	TRUE	0.38	0.44	-0.06	0.037
cg24083641	*MGC46496*	4	TRUE	0.40	0.45	-0.06	0.013

cg02586730	*EFEMP2*	11	TRUE	0.38	0.44	-0.06	0.044
cg05856931	*MSI2*	17	TRUE	0.52	0.57	-0.06	0.003
cg07425555	*FLJ23447*	19	FALSE	0.27	0.33	-0.06	0.002
cg24450312	*RASSF5*	1	TRUE	0.16	0.22	-0.06	0.001
cg14892768	*AXL*	19	FALSE	0.16	0.21	-0.06	0.008
cg03942271	*AUH*	9	TRUE	0.26	0.32	-0.06	0.019
cg14191360	*MET*	7	TRUE	0.60	0.66	-0.06	0.047
cg03532005	*PSPH*	7	TRUE	0.24	0.30	-0.06	0.002
cg11503011	*EBF*	5	FALSE	0.16	0.22	-0.06	0.001
cg05179880	*PDCD7*	15	TRUE	0.22	0.28	-0.06	0.021
cg00066816	*IL12B*	5	TRUE	0.35	0.41	-0.06	0.029
cg04759439	*CAST1*	3	TRUE	0.19	0.25	-0.06	<0.001
cg04065065	*PIGO*	9	TRUE	0.20	0.26	-0.06	<0.001
cg11976166	*TGFB2*	1	TRUE	0.19	0.25	-0.06	0.001
cg08697665	*RARRES1*	3	TRUE	0.33	0.39	-0.06	0.011
cg22134325	*NPAS4*	11	TRUE	0.17	0.23	-0.06	0.012
cg08216808	*ARPC2*	2	FALSE	0.22	0.28	-0.06	0.009
cg14166009	*HKR1*	19	TRUE	0.18	0.23	-0.06	0.013
cg25482967	*MRPS10*	6	TRUE	0.31	0.36	-0.06	0.010
cg02214188	*BDH2*	4	FALSE	0.47	0.52	-0.06	0.004
cg16152813	*FAM57B*	16	FALSE	0.16	0.22	-0.06	0.001
cg11466908	*AUH*	9	TRUE	0.09	0.15	-0.06	<0.001
cg04371779	*GPATCH2L*	14	TRUE	0.09	0.15	-0.06	0.008
cg02401978	*ZNF746*	7	FALSE	0.53	0.58	-0.06	0.040
cg01457653	*FBXL8*	16	TRUE	0.09	0.14	-0.06	0.024
cg09310112	*JMJD2B*	19	TRUE	0.09	0.15	-0.06	0.022
cg11418559	*ZNF593*	1	TRUE	0.27	0.33	-0.06	0.025
cg27069753	*ELA3B*	1	FALSE	0.51	0.57	-0.06	0.020
cg17593391	*RRAGC*	1	TRUE	0.26	0.32	-0.06	0.046
cg08749917	*RTP1*	3	FALSE	0.43	0.49	-0.06	0.029
cg07036530	*GPR26*	10	TRUE	0.29	0.35	-0.06	0.012
cg21151355	*GJB3*	1	FALSE	0.24	0.30	-0.06	0.008
cg17166338	*TERT*	5	TRUE	0.48	0.54	-0.06	0.026
cg19350340	*ASPM*	1	TRUE	0.32	0.38	-0.06	0.008
cg22986999	*MRGPRF*	11	TRUE	0.28	0.34	-0.06	0.042
cg27112247	*TMED2*	12	TRUE	0.53	0.59	-0.06	<0.001
cg01200177	*POLR2D*	2	TRUE	0.31	0.37	-0.06	0.011
cg11052143	*ALS2CR11*	2	TRUE	0.18	0.25	-0.06	0.025
cg03682712	*LOXL1*	15	TRUE	0.10	0.16	-0.06	0.016

cg20119051	*R3HDM1*	2	TRUE	0.48	0.54	-0.06	0.001
cg10927536	*SRRD*	22	TRUE	0.35	0.41	-0.06	0.019
cg04845628	*MINA*	3	TRUE	0.63	0.69	-0.06	0.001
cg05194726	*NRIP2*	12	FALSE	0.11	0.17	-0.06	0.017
cg11412582	*HERC2*	15	TRUE	0.12	0.18	-0.06	0.024
cg23769143	*TIMP4*	3	FALSE	0.18	0.25	-0.06	0.032
cg12256080	*ABT1*	6	TRUE	0.09	0.15	-0.06	0.004
cg11730100	*FEN1*	11	TRUE	0.23	0.29	-0.06	0.002
cg25225238	*GRM3*	7	FALSE	0.33	0.39	-0.06	0.039
cg12324629	*ULK2*	17	TRUE	0.37	0.44	-0.06	0.029
cg04532952	*CA4*	17	TRUE	0.14	0.20	-0.06	0.002
cg15557833	*MFSD8*	4	TRUE	0.25	0.32	-0.06	<0.001
cg07884019	*HMP19*	5	FALSE	0.28	0.34	-0.06	0.047
cg11177693	*ZNF513*	2	TRUE	0.56	0.62	-0.06	0.020
cg21759080	*STX5A*	11	TRUE	0.13	0.19	-0.06	0.021
cg24674220	*GMEB2*	20	FALSE	0.71	0.77	-0.06	0.045
cg03098721	*TTLL7*	1	TRUE	0.58	0.64	-0.06	0.024
cg25982743	*TIMP4*	3	TRUE	0.14	0.21	-0.06	0.001
cg20856064	*PH-4*	3	TRUE	0.17	0.23	-0.06	<0.001
cg24792360	*FUCA1*	1	TRUE	0.41	0.48	-0.06	0.038
cg03513163	*PCDHB1*	5	TRUE	0.29	0.35	-0.06	0.032
cg05587474	*MOV10*	1	TRUE	0.38	0.45	-0.06	<0.001
cg17327630	*ZNF212*	7	TRUE	0.17	0.23	-0.07	0.007
cg00647741	*ZNF274*	19	FALSE	0.43	0.49	-0.07	0.043
cg11536940	*PGCP*	8	TRUE	0.25	0.32	-0.07	0.038
cg17865752	*CCNT2*	2	TRUE	0.30	0.36	-0.07	0.019
cg19974223	*ANKRD33*	12	TRUE	0.60	0.66	-0.07	0.013
cg21493666	*PPME1*	11	TRUE	0.20	0.26	-0.07	0.005
cg05303448	*AACS*	12	TRUE	0.22	0.29	-0.07	0.001
cg10994379	*CAPSL*	5	FALSE	0.37	0.44	-0.07	0.050
cg00571634	*WDR5B*	3	TRUE	0.41	0.48	-0.07	0.003
cg14920846	*NAV1*	1	TRUE	0.42	0.48	-0.07	0.025
cg08707819	*RCOR1*	14	TRUE	0.35	0.42	-0.07	0.001
cg24362726	*C4orf33*	4	FALSE	0.64	0.71	-0.07	0.048
cg26431343	*HSPC117*	22	FALSE	0.48	0.55	-0.07	0.005
cg11908570	*KIAA0040*	1	TRUE	0.24	0.31	-0.07	0.001
cg22972055	*UNC84A*	7	TRUE	0.68	0.75	-0.07	0.019
cg17783509	*PHOX2B*	4	FALSE	0.30	0.37	-0.07	0.009
cg23547429	*SLC43A3*	11	FALSE	0.20	0.26	-0.07	<0.001

cg14435807	*LOXL1*	15	TRUE	0.13	0.20	-0.07	0.002
cg05257610	*NDUFAF7*	2	FALSE	0.57	0.64	-0.07	0.039
cg01860753	*RASSF5*	1	TRUE	0.22	0.29	-0.07	0.012
cg12271671	*FGL2*	7	FALSE	0.29	0.35	-0.07	0.016
cg07129274	*HIST1H2AL*	6	TRUE	0.27	0.34	-0.07	0.012
cg02860543	*IGF2BP3*	7	TRUE	0.42	0.49	-0.07	0.043
cg25345738	*PWP1*	12	FALSE	0.16	0.22	-0.07	0.018
cg01718365	*AOF2*	1	TRUE	0.24	0.31	-0.07	0.008
cg01288089	*CXCL10*	4	FALSE	0.25	0.32	-0.07	<0.001
cg02147791	*SAP18*	13	TRUE	0.22	0.29	-0.07	0.035
cg09404633	*LMOD1*	1	TRUE	0.21	0.28	-0.07	0.001
cg15868302	*FOXD2*	1	TRUE	0.42	0.49	-0.07	0.018
cg09047884	*TTLL1*	22	TRUE	0.58	0.65	-0.07	0.001
cg02849695	*CCDC19*	1	TRUE	0.24	0.31	-0.07	0.001
cg07693270	*RPL39L*	3	TRUE	0.41	0.48	-0.07	<0.001
cg03160508	*RHOD*	11	TRUE	0.18	0.25	-0.07	0.015
cg18632102	*CNOT8*	5	TRUE	0.37	0.44	-0.07	0.020
cg20426860	*FLJ20422*	19	TRUE	0.18	0.25	-0.07	0.006
cg24088408	*E2F1*	20	TRUE	0.53	0.61	-0.07	0.027
cg21422208	*RFT1*	3	TRUE	0.28	0.35	-0.07	0.011
cg19453250	*STATIP1*	18	FALSE	0.31	0.38	-0.07	0.007
cg18847227	*SUMF1*	3	TRUE	0.39	0.47	-0.07	0.015
cg27239157	*MCF2L2*	3	TRUE	0.41	0.48	-0.07	<0.001
cg15815843	*MFAP5*	12	FALSE	0.72	0.79	-0.07	0.017
cg20773127	*ENPEP*	4	TRUE	0.41	0.48	-0.07	0.037
cg03876622	*CSDA*	12	TRUE	0.21	0.29	-0.07	0.017
cg14036856	*MGC52423*	1	FALSE	0.62	0.69	-0.07	0.025
cg17387870	*CHFR*	12	TRUE	0.24	0.31	-0.07	0.017
cg11672225	*RNF185*	22	TRUE	0.20	0.27	-0.07	0.006
cg01337047	*DSG1*	18	FALSE	0.46	0.53	-0.07	0.048
cg08713365	*C20orf98*	20	TRUE	0.08	0.16	-0.07	0.017
cg14711201	*SKP2*	5	TRUE	0.14	0.21	-0.07	0.001
cg07460665	*CALCOCO2*	17	TRUE	0.16	0.23	-0.07	0.002
cg19759064	*PHKG1*	7	FALSE	0.29	0.36	-0.07	0.046
cg11816577	*YWHAE*	17	TRUE	0.15	0.23	-0.07	0.047
cg18977436	*FGF14*	13	FALSE	0.32	0.39	-0.08	0.006
cg26924825	*LCAT*	16	FALSE	0.54	0.62	-0.08	0.039
cg15881727	*ZBED4*	22	TRUE	0.11	0.18	-0.08	0.001
cg00674922	*SATB1*	3	TRUE	0.39	0.46	-0.08	0.005

cg05624932	*CRISPLD1*	8	TRUE	0.35	0.42	-0.08	0.031
cg17378989	*ERCC1*	19	TRUE	0.32	0.40	-0.08	0.020
cg27394046	*XRN2*	20	TRUE	0.51	0.59	-0.08	0.027
cg05333568	*CCDC185*	1	TRUE	0.26	0.34	-0.08	0.019
cg16121444	*NME7*	1	TRUE	0.16	0.23	-0.08	0.025
cg01919208	*LAMB2*	3	TRUE	0.16	0.24	-0.08	0.009
cg20055101	*ZNF206*	16	FALSE	0.33	0.41	-0.08	0.041
cg10217449	*GNPDA2*	4	TRUE	0.45	0.53	-0.08	0.018
cg18964732	*TMCO1*	1	TRUE	0.16	0.24	-0.08	0.002
cg21993406	*CENPH*	5	FALSE	0.32	0.40	-0.08	0.039
cg01446393	*FAM107A*	3	FALSE	0.58	0.66	-0.08	0.045
cg26284390	*NFKBIZ*	3	TRUE	0.25	0.33	-0.08	0.026
cg13549845	*GRID2*	4	TRUE	0.48	0.56	-0.08	0.011
cg00984602	*SRF*	6	TRUE	0.20	0.28	-0.08	0.001
cg26486702	*USP21*	1	TRUE	0.23	0.31	-0.08	0.022
cg05420896	*DCC*	18	TRUE	0.29	0.37	-0.08	0.007
cg10841258	*CTTNBP2NL*	1	TRUE	0.23	0.31	-0.08	0.037
cg12148581	*RPL14*	3	TRUE	0.41	0.49	-0.08	0.028
cg18053607	*PIB5PA*	22	FALSE	0.22	0.30	-0.08	0.045
cg12435611	*BRIP1*	17	TRUE	0.28	0.36	-0.08	0.014
cg19972619	*MYC*	8	TRUE	0.08	0.16	-0.08	0.005
cg24333473	*ZNF597*	16	TRUE	0.23	0.31	-0.08	0.032
cg05681757	*FGD4*	12	FALSE	0.34	0.43	-0.09	0.001
cg16858125	*ELOVL1*	1	TRUE	0.45	0.54	-0.09	0.018
cg21374864	*TOR1A*	9	TRUE	0.30	0.39	-0.09	0.012
cg06363129	*SOSTDC1*	7	FALSE	0.34	0.42	-0.09	0.001
cg03813905	*NDUFB9*	8	TRUE	0.28	0.37	-0.09	0.011
cg01185080	*ZNF710*	15	TRUE	0.25	0.34	-0.09	0.005
cg25162921	*MTAP*	9	TRUE	0.24	0.33	-0.09	0.004
cg23904249	*MGC2574*	11	TRUE	0.59	0.68	-0.09	0.001
cg08005849	*HGF*	7	FALSE	0.38	0.47	-0.09	0.005
cg12477119	*CRKRS*	17	TRUE	0.30	0.39	-0.09	0.003
cg04561804	*TLOC1*	3	FALSE	0.30	0.39	-0.09	0.025
cg09494546	*SLC16A4*	1	FALSE	0.58	0.67	-0.09	0.017
cg24115040	*DLX5*	7	TRUE	0.42	0.51	-0.09	0.021
cg16706631	*HIST1H4E*	6	TRUE	0.29	0.38	-0.09	0.038
cg06609049	*THOP1*	19	TRUE	0.36	0.45	-0.09	0.016
cg08586737	*GCC1*	7	TRUE	0.30	0.39	-0.09	0.038
cg27546682	*STK40*	1	TRUE	0.29	0.38	-0.09	0.004

cg15298323	*ACAT2*	6	TRUE	0.62	0.71	-0.09	0.001
cg01745657	*PLCXD2*	3	TRUE	0.28	0.37	-0.09	0.005
cg04975920	*TSPAN1*	1	FALSE	0.47	0.56	-0.09	0.047
cg20050113	*SLC9A2*	2	TRUE	0.35	0.44	-0.09	0.016
cg03787486	*ADH5*	4	TRUE	0.24	0.34	-0.09	<0.001
cg01985396	*DAAM2*	6	FALSE	0.26	0.35	-0.10	<0.001
cg15780361	*ALS2CR11*	2	TRUE	0.33	0.43	-0.10	0.006
cg22496254	*HCAP-G*	4	FALSE	0.56	0.65	-0.10	0.035
cg23242898	*DCC*	18	TRUE	0.57	0.67	-0.10	0.011
cg06493080	*HOXB7*	17	TRUE	0.35	0.45	-0.10	0.038
cg10735607	*TMEM109*	11	TRUE	0.53	0.63	-0.10	0.048
cg19573166	*SLC22A17*	14	FALSE	0.47	0.57	-0.10	0.035
cg27413025	*CYP20A1*	2	TRUE	0.28	0.38	-0.10	<0.001
cg24745738	*EDNRB*	13	TRUE	0.22	0.32	-0.10	0.001
cg10003443	*FOXA2*	20	TRUE	0.36	0.46	-0.10	0.010
cg07455279	*NDUFA3*	19	TRUE	0.21	0.31	-0.10	0.046
cg27202708	*CCDC185*	1	TRUE	0.27	0.38	-0.11	0.001
cg03882305	*TRIM50C*	7	TRUE	0.38	0.49	-0.11	0.003
cg26777475	*PCOLCE*	7	FALSE	0.44	0.55	-0.11	0.048
cg04689061	*PKIA*	8	TRUE	0.31	0.42	-0.11	0.025
cg03454353	*ZFP37*	9	TRUE	0.45	0.56	-0.11	<0.001
cg20229788	*GCKR*	2	FALSE	0.37	0.49	-0.11	0.005
cg23970338	*TSFM*	12	TRUE	0.13	0.25	-0.12	0.010
cg21168622	*ZNF350*	19	TRUE	0.24	0.36	-0.12	0.032
cg22502502	*TRIM38*	6	FALSE	0.28	0.40	-0.12	0.002
cg08965324	*LTA4H*	12	TRUE	0.32	0.45	-0.13	0.032
cg20587336	*ARMC1*	8	TRUE	0.35	0.49	-0.14	0.006
cg07713361	*APOL1*	22	FALSE	0.41	0.55	-0.14	0.003
cg12992720	*EDG4*	19	TRUE	0.25	0.39	-0.14	0.001
cg25125453	*SPTLC3*	20	FALSE	0.44	0.58	-0.15	0.002
cg08942800	*CRISP2*	6	TRUE	0.36	0.52	-0.17	0.017

Table S4. List of DMCs in LT in the comparison between the DO and NDO groups

Target ID	Symbol	CHR	CPG Island	Beta DO	Beta OB	Delta Beta	*p*-value
cg10296238	*SPATC1L*	21	TRUE	0.59	0.47	0.12	0.029
cg18145505	*GREM1*	15	TRUE	0.35	0.23	0.12	0.013
cg23002761	*FBLIM1*	1	TRUE	0.31	0.19	0.11	0.026
cg10746737	*HLA-DRB5*	6	FALSE	0.71	0.61	0.11	0.018
cg09794131	*HYDIN*	16	TRUE	0.34	0.23	0.11	0.030
cg07747299	*SPATC1L*	21	TRUE	0.56	0.46	0.10	0.040
cg06851207	*FLJ10781*	19	TRUE	0.72	0.62	0.09	0.028
cg06361108	*CCNF*	16	TRUE	0.21	0.13	0.09	0.009
cg25902889	*FSD1*	19	FALSE	0.62	0.53	0.09	0.009
cg17483510	*GNB4*	3	TRUE	0.45	0.36	0.08	0.011
cg26764244	*GNG12*	1	TRUE	0.28	0.20	0.08	0.034
cg16670497	*GSTM2*	1	TRUE	0.44	0.36	0.08	0.024
cg17179881	*BIK*	22	TRUE	0.62	0.54	0.08	0.017
cg12699145	*TMC7*	16	TRUE	0.29	0.21	0.08	0.009
cg07404485	*PON1*	7	FALSE	0.38	0.31	0.08	0.002
cg06268694	*CELSR1*	22	TRUE	0.30	0.23	0.07	0.026
cg26577529	*KRT6E*	12	FALSE	0.73	0.65	0.07	0.016
cg04557383	*MT1H*	16	TRUE	0.43	0.35	0.07	0.005
cg19297232	*SMPD3*	16	TRUE	0.59	0.52	0.07	0.029
cg22039287	*RIT2*	18	FALSE	0.61	0.53	0.07	0.006
cg22609784	*MSX1*	4	TRUE	0.55	0.48	0.07	0.001
cg07640473	*SEMA3F*	3	TRUE	0.51	0.44	0.07	0.029
cg10447080	*FILIP1*	6	FALSE	0.37	0.30	0.07	0.012
cg18055394	*EPHA3*	3	TRUE	0.34	0.27	0.07	0.041
cg03283421	*CUZD1*	10	FALSE	0.72	0.65	0.07	0.034
cg08137040	*ZNF800*	7	TRUE	0.35	0.28	0.07	0.003
cg15534366	*CDH4*	20	FALSE	0.52	0.46	0.07	0.012
cg00563926	*TGFBR3*	1	TRUE	0.27	0.21	0.06	0.047
cg26525091	*MADCAM1*	19	TRUE	0.24	0.18	0.06	0.004
cg00949442	*ABCA3*	16	TRUE	0.24	0.18	0.06	0.002
cg06147863	*SPI1*	11	FALSE	0.67	0.61	0.06	0.001
cg07914866	*IRAK3*	12	TRUE	0.33	0.27	0.06	0.010
cg13391638	*FLJ13576*	7	TRUE	0.41	0.35	0.06	0.028
cg21974239	*MAPK12*	22	TRUE	0.22	0.16	0.06	0.007
cg00489401	*FLT4*	5	TRUE	0.20	0.14	0.06	0.021
cg15778232	*PHB2*	12	FALSE	0.63	0.57	0.06	0.019

cg13975369	*TSGA14*	7	TRUE	0.32	0.26	0.06	0.010
cg14620221	*OR8B8*	11	FALSE	0.80	0.74	0.06	0.018
cg08946332	*ALOX12*	17	TRUE	0.87	0.81	0.06	0.016
cg27038439	*MSX1*	4	TRUE	0.75	0.70	0.06	0.044
cg12499211	*SH2D2A*	1	TRUE	0.44	0.38	0.06	0.049
cg24789869	*DDX11*	12	TRUE	0.12	0.07	0.06	0.001
cg26069745	*HOXA2*	7	TRUE	0.25	0.19	0.06	0.021
cg27063525	*NUS1*	6	TRUE	0.17	0.11	0.06	0.049
cg03760483	*ALOX12*	17	TRUE	0.43	0.37	0.06	0.043
cg18815943	*FOXE3*	1	TRUE	0.13	0.07	0.06	0.001
cg07637239	*KCNK18*	10	FALSE	0.52	0.46	0.06	0.018
cg09328024	*DYRK3*	1	TRUE	0.28	0.22	0.06	0.022
cg00043004	*NOXO1*	16	TRUE	0.16	0.11	0.06	0.006
cg18501026	*DOCK3*	3	TRUE	0.23	0.17	0.06	0.002
cg19777470	*CRABP1*	15	TRUE	0.25	0.20	0.06	0.013
cg13707560	*NME5*	5	TRUE	0.42	0.37	0.05	0.026
cg09606564	*MFAP4*	17	FALSE	0.34	0.29	0.05	0.036
cg19428417	*RRAD*	16	TRUE	0.52	0.47	0.05	0.025
cg09238677	*C3AR1*	12	FALSE	0.47	0.41	0.05	0.047
cg00328227	*C1orf59*	1	TRUE	0.53	0.48	0.05	0.048
cg16352283	*FAM46B*	1	TRUE	0.52	0.47	0.05	0.031
cg21096915	*MGC16291*	10	TRUE	0.17	0.22	-0.05	0.013
cg19149785	*KLK8*	19	TRUE	0.65	0.70	-0.05	0.044
cg16330965	*SNAPC5*	15	TRUE	0.23	0.28	-0.05	0.012
cg06690548	*SLC7A11*	4	TRUE	0.73	0.78	-0.05	0.005
cg13817266	*HSPA6*	1	TRUE	0.35	0.40	-0.05	0.047
cg02104644	*SYT7*	11	TRUE	0.11	0.16	-0.05	0.046
cg10735607	*TMEM109*	11	TRUE	0.48	0.53	-0.05	0.013
cg15619125	*MAMDC4*	9	FALSE	0.44	0.49	-0.05	0.037
cg00754253	*HRASLS5*	11	FALSE	0.33	0.38	-0.05	0.035
cg13823701	*TNXB*	6	FALSE	0.27	0.33	-0.05	0.049
cg26125600	*PF4V1*	4	FALSE	0.38	0.43	-0.05	0.003
cg26205432	*PLN*	6	FALSE	0.56	0.61	-0.05	0.032
cg15784615	*LTBR*	12	FALSE	0.22	0.27	-0.05	0.013
cg14861570	*MMD*	17	FALSE	0.26	0.32	-0.05	0.001
cg09547190	*C9orf89*	9	TRUE	0.38	0.44	-0.05	0.009
cg23685580	*CATSPER2*	15	TRUE	0.81	0.87	-0.05	0.027
cg18940763	*XBP1*	22	TRUE	0.13	0.19	-0.05	0.013
cg05341115	*HYAL4*	7	FALSE	0.67	0.72	-0.05	0.001

cg14532519	*TCF20*	22	FALSE	0.58	0.63	-0.05	0.019
cg24603941	*MIA2*	14	FALSE	0.51	0.57	-0.05	0.023
cg12815142	*SPAG7*	17	TRUE	0.30	0.36	-0.05	0.037
cg04991214	*PFDN2*	1	FALSE	0.50	0.56	-0.05	0.050
cg14882700	*OTOP1*	4	TRUE	0.20	0.26	-0.05	0.037
cg06948408	*TMEM125*	1	TRUE	0.18	0.23	-0.05	0.017
cg18184219	*CEP170*	1	FALSE	0.73	0.79	-0.05	0.031
cg02741177	*PROL1*	4	FALSE	0.65	0.70	-0.05	0.005
cg03964111	*LR8*	7	FALSE	0.17	0.22	-0.05	0.003
cg17122311	*IL27*	16	FALSE	0.46	0.51	-0.05	0.040
cg10742801	*PRAP1*	10	FALSE	0.29	0.34	-0.05	0.006
cg01035422	*PLIN*	15	FALSE	0.70	0.76	-0.05	0.018
cg01895214	*LOC654342*	2	TRUE	0.49	0.54	-0.05	0.001
cg01040850	*MR1*	1	FALSE	0.53	0.59	-0.05	0.046
cg14851685	*CYP4F22*	19	FALSE	0.60	0.65	-0.05	0.036
cg05507459	*NXNL2*	9	TRUE	0.29	0.34	-0.05	0.001
cg00135393	*FGG*	4	FALSE	0.31	0.37	-0.05	0.036
cg25017250	*APOC4*	19	TRUE	0.28	0.34	-0.05	0.038
cg13431205	*RB1*	13	TRUE	0.51	0.57	-0.05	0.039
cg10574499	*UNQ2446*	16	FALSE	0.28	0.34	-0.06	0.037
cg02655623	*HSA277841*	17	FALSE	0.61	0.67	-0.06	0.003
cg03894103	*PREPL*	2	FALSE	0.70	0.76	-0.06	0.033
cg07514381	*LRRC2*	3	FALSE	0.69	0.75	-0.06	0.042
cg14477619	*NPC1L1*	7	FALSE	0.41	0.47	-0.06	0.013
cg04106785	*CDK5R1*	17	TRUE	0.32	0.37	-0.06	0.010
cg17229388	*MGC35169*	13	TRUE	0.70	0.76	-0.06	0.022
cg13849691	*ACSL5*	10	TRUE	0.78	0.84	-0.06	0.050
cg06048973	*ACTC*	15	FALSE	0.50	0.55	-0.06	0.044
cg08137716	*TTLL6*	17	FALSE	0.71	0.76	-0.06	0.022
cg24652919	*WDR58*	16	FALSE	0.64	0.69	-0.06	0.039
cg20199333	*F2*	11	FALSE	0.48	0.54	-0.06	0.016
cg24898863	*S100A8*	1	FALSE	0.23	0.29	-0.06	0.002
cg20099806	*CCDC47*	17	FALSE	0.25	0.31	-0.06	0.004
cg18294158	*ORC5L*	7	FALSE	0.66	0.72	-0.06	0.007
cg15398520	*LPAL2*	6	FALSE	0.35	0.41	-0.06	0.027
cg25677709	*NDST1*	5	FALSE	0.50	0.56	-0.06	0.016
cg24269657	*F7*	13	FALSE	0.41	0.46	-0.06	0.006
cg00466492	*CTXN1*	19	TRUE	0.76	0.81	-0.06	0.025
cg01484156	*NCALD*	8	FALSE	0.34	0.40	-0.06	<0.001

cg20584011	*ZDHHC11*	5	TRUE	0.72	0.78	-0.06	0.002
cg16153267	*RB1*	13	TRUE	0.56	0.61	-0.06	0.008
cg22337624	*DHX38*	16	FALSE	0.27	0.33	-0.06	0.003
cg06850526	*MGC15523*	17	TRUE	0.27	0.33	-0.06	0.039
cg18901980	*KRT25A*	17	FALSE	0.68	0.74	-0.06	0.003
cg16639185	*LGTN*	1	TRUE	0.33	0.39	-0.06	0.031
cg10303487	*DPYS*	8	TRUE	0.07	0.13	-0.06	0.033
cg00579402	*FUT6*	19	FALSE	0.52	0.58	-0.06	0.034
cg04837071	*NOXA1*	9	TRUE	0.53	0.59	-0.06	0.033
cg12694870	*HPD*	12	FALSE	0.38	0.44	-0.06	0.027
cg07233761	*ESM1*	5	TRUE	0.27	0.33	-0.06	0.031
cg22171829	*PDK4*	7	TRUE	0.12	0.18	-0.06	0.037
cg15201291	*CYP2C8*	10	FALSE	0.53	0.60	-0.06	0.014
cg05985767	*ANPEP*	15	TRUE	0.36	0.42	-0.06	0.008
cg07163603	*HLA-A*	6	FALSE	0.34	0.40	-0.06	0.032
cg24920358	*PPIE*	1	TRUE	0.32	0.38	-0.06	0.046
cg03835296	*SLC17A1*	6	FALSE	0.32	0.38	-0.07	0.014
cg12542604	*ANKS1A*	6	TRUE	0.14	0.20	-0.07	0.020
cg07745725	*PSG3*	19	FALSE	0.55	0.62	-0.07	0.008
cg02523400	*SERPIND1*	22	FALSE	0.34	0.40	-0.07	0.048
cg24792360	*FUCA1*	1	TRUE	0.34	0.41	-0.07	0.011
cg27420123	*FSHB*	11	FALSE	0.34	0.41	-0.07	0.007
cg23834593	*HNF4A*	20	FALSE	0.37	0.44	-0.07	0.029
cg10414946	*MS4A2*	11	FALSE	0.47	0.54	-0.07	0.020
cg07150830	*NOS2A*	17	FALSE	0.52	0.59	-0.07	0.020
cg25514304	*PSEN2*	1	FALSE	0.42	0.49	-0.07	0.041
cg15783800	*HAK*	18	TRUE	0.36	0.43	-0.07	0.007
cg27440834	*SNX4*	3	FALSE	0.56	0.63	-0.07	0.038
cg02735486	*ANK2*	4	TRUE	0.46	0.53	-0.07	0.030
cg15149645	*P8*	16	FALSE	0.17	0.24	-0.07	0.003
cg13614083	*KCNAB2*	1	TRUE	0.51	0.58	-0.07	0.032
cg12003230	*LINC00313*	21	FALSE	0.48	0.55	-0.07	0.017
cg02399455	*SRI*	7	FALSE	0.54	0.61	-0.07	0.025
cg15357639	*OGG1*	3	TRUE	0.78	0.85	-0.07	0.013
cg07109801	*NDUFAF3*	3	TRUE	0.50	0.57	-0.07	0.018
cg19464944	*FCGR1A*	1	FALSE	0.31	0.39	-0.07	0.038
cg20615832	*PF4V1*	4	TRUE	0.28	0.35	-0.07	0.022
cg23322523	*TRIM55*	8	FALSE	0.26	0.33	-0.07	0.013
cg05976325	*LOC284912*	22	FALSE	0.51	0.58	-0.07	0.038

cg13520715	*LINC00479*	21	FALSE	0.70	0.77	-0.07	0.022
cg25657700	*SNRPN*	15	TRUE	0.54	0.62	-0.07	0.026
cg04994456	*RNF186*	1	FALSE	0.40	0.48	-0.08	0.010
cg19554294	*VN1R2*	19	FALSE	0.51	0.58	-0.08	0.010
cg18429742	*ZDHHC11*	5	TRUE	0.64	0.72	-0.08	0.016
cg13705284	*ACOX2*	3	FALSE	0.19	0.27	-0.08	0.012
cg13726463	*COX6A2*	16	TRUE	0.51	0.58	-0.08	0.004
cg26955850	*OXT*	20	TRUE	0.37	0.45	-0.08	0.050
cg04662594	*EPB49*	8	FALSE	0.32	0.40	-0.08	0.002
cg20131968	*CCDC47*	17	FALSE	0.33	0.42	-0.08	0.020
cg03743584	*PRAP1*	10	FALSE	0.19	0.27	-0.08	0.003
cg06784466	*FPRL2*	19	FALSE	0.39	0.48	-0.08	0.008
cg11108890	*VAMP5*	2	TRUE	0.10	0.18	-0.08	0.040
cg26631477	*GPR125*	4	TRUE	0.19	0.27	-0.08	<0.001
cg02142461	*LYAR*	4	FALSE	0.72	0.80	-0.08	0.013
cg03533858	*MORN1*	1	FALSE	0.43	0.51	-0.08	0.029
cg08510456	*BSN*	3	TRUE	0.39	0.47	-0.08	0.039
cg06194808	*MGC9712*	7	FALSE	0.48	0.57	-0.09	0.008
cg25598083	*ACOT2*	14	FALSE	0.48	0.57	-0.09	0.025
cg07251788	*CLTCL1*	22	TRUE	0.42	0.51	-0.09	0.028
cg18678121	*SEC61A2*	10	TRUE	0.50	0.60	-0.09	0.011
cg03733371	*LIPH*	3	FALSE	0.44	0.54	-0.10	0.047
cg02192965	*SLC3A1*	2	FALSE	0.60	0.70	-0.10	0.023
cg24333473	*ZNF597*	16	TRUE	0.37	0.47	-0.10	0.042
cg17264470	*FGF21*	19	FALSE	0.76	0.87	-0.11	0.016
cg22730830	*PRSS21*	16	TRUE	0.57	0.68	-0.11	0.003
cg24362726	*C4orf33*	4	FALSE	0.31	0.42	-0.11	0.039
cg01600189	*FLJ20444*	9	TRUE	0.18	0.30	-0.11	0.005
cg24092914	*VHL*	3	TRUE	0.46	0.58	-0.12	0.021
cg26267561	*OXT*	20	TRUE	0.68	0.84	-0.15	0.001
cg20655558	*DNAJB7*	22	FALSE	0.56	0.72	-0.16	0.035
cg23349790	*IGSF21*	1	TRUE	0.12	0.30	-0.18	<0.001

Table S5 . Gene ontology enrichment analysis using the genes with DMCs in SAT

GO term	Fold enrichment	Count	*p* value	Genes
Regulation of cellular ketone metabolic process	22.1	4	0.001	*LEP, CPT1B, IRS1, ADIPOQ*
Regulation of carbohydrate biosynthetic process	44.9	3	0.002	*LEP, IRS1, ADIPOQ*
Muscle organ development	7.5	5	0.004	*CPT1B, TSC1, MYF5, SGCD, ALX4*
Regulation of lipid metabolic process	11.2	4	0.005	*LEP, CPT1B, IRS1, ADIPOQ*
Regulation of glucose metabolic process	27	3	0.005	*LEP, IRS1, ADIPOQ*
Fatty acid catabolic process	26.2	3	0.006	*LEP, CPT1B, ADIPOQ*
Regulation of cellular carbohydrate metabolic process	24.8	3	0.006	*LEP, IRS1, ADIPOQ*
Regulation of carbohydrate metabolic process	24.2	3	0.006	*LEP, IRS1, ADIPOQ*
Regulation of fatty acid metabolic process	19.3	3	0.010	*CPT1B, IRS1, ADIPOQ*
Embryonic skeletal system morphogenesis	16.6	3	0.013	*HOXA4, MYF5, ALX4*
Skeletal system development	4.9	5	0.017	*HOXA4, CLEC3A, KAZALD1, MYF5, ALX4*
Development of primary female sexual characteristics	13.7	3	0.019	*LEP, FGF7, FANCG*
Cell adhesion	3.1	7	0.020	*TSC1, MYF5, SIGLEC7, LGALS7, NINJ2, CTNNA1, DST*
Biological adhesion	3.1	7	0.020	*TSC1, MYF5, SIGLEC7, LGALS7, NINJ2, CTNNA1, DST*
Cellular lipid catabolic process	12.4	3	0.023	*LEP, CPT1B, ADIPOQ*
Regulation of gluconeogenesis	78.7	2	0.025	*LEP, ADIPOQ*
Negative regulation of response to stimulus	9.4	3	0.038	*LEP, IRS1, ADIPOQ*
Response to insulin stimulus	9.4	3	0.038	*LEP, TSC1, IRS1*
Phosphatidylcholine biosynthetic process	44.9	2	0.043	*CPT1B, FGF7*
Organic acid catabolic process	8.5	3	0.046	*LEP, CPT1B, ADIPOQ*
Carboxylic acid catabolic process	8.5	3	0.046	*LEP, CPT1B, ADIPOQ*
Skeletal system morphogenesis	8.4	3	0.047	*HOXA4, MYF5, ALX4*

Table S6 . Gene ontology enrichment analysis using the genes with DMCs in VAT

GO term	Fold enrichment	Count	*p* value	Genes
Regulation of apoptosis	1.9	26	0.003	*DCC, MEF2C, ADA, TGFB2, EDNRB, ATG5, PAX7, GRID2, TNFRSF19, MYC, TERT, ERCC1, FGD4, CD3E, SKP2, TRIO, HGF, CSDA, YWHAE, BRCA1, JMY, KCNH8, IL12B, TIAF1, PDCD7, DNM2*
Regulation of programmed cell death	1.9	26	0.003	*DCC, MEF2C, ADA, TGFB2, EDNRB, ATG5, PAX7, GRID2, TNFRSF19, MYC, TERT, ERCC1, FGD4, CD3E, SKP2, TRIO, HGF, CSDA, YWHAE, BRCA1, JMY, KCNH8, IL12B, TIAF1, PDCD7, DNM2*
Regulation of cell death	1.9	26	0.003	*DCC, MEF2C, ADA, TGFB2, EDNRB, ATG5, PAX7, GRID2, TNFRSF19, MYC, TERT, ERCC1, FGD4, CD3E, SKP2, TRIO, HGF, CSDA, YWHAE, BRCA1, JMY, KCNH8, IL12B, TIAF1, PDCD7, DNM2*
Negative regulation of lyase activity	5.3	5	0.014	*EDNRB, GRM3, ADCY7, GALR1, GRM6*
Negative regulation of adenylate cyclase activity	5.3	5	0.014	*EDNRB, GRM3, ADCY7, GALR1, GRM6*
Negative regulation of cyclase activity	5.3	5	0.014	*EDNRB, GRM3, ADCY7, GALR1, GRM6*
Negative regulation of neuron differentiation	7.1	4	0.018	*PHOX2B, FOXA2, DLL1, ASPM*
Negative regulation of apoptosis	2.1	13	0.019	*MEF2C, SKP2, HGF, CSDA, ADA, EDNRB, ATG5, PAX7, KCNH8, TIAF1, MYC, ERCC1, TERT*
DNA catabolic process	4.9	5	0.019	*GTF2H4, DNASE1L2, MYC, XRN2, ERCC1*
Negative regulation of programmed cell death	2.1	13	0.02	*MEF2C, SKP2, HGF, CSDA, ADA, EDNRB, ATG5, PAX7, KCNH8, TIAF1, MYC, ERCC1, TERT*
Negative regulation of cell death	2.1	13	0.021	*MEF2C, SKP2, HGF, CSDA, ADA, EDNRB, ATG5, PAX7, KCNH8, TIAF1, MYC, ERCC1, TERT*
Regulation of adenylate cyclase activity	3.6	6	0.024	*EDNRB, GRM3, ADCY7, GALR1, GRM6, GNAS*
Regulation of cell cycle	2.1	12	0.027	*E2F1, CCNT2, SKP2, RPRM, BRIP1, NUSAP1, HERC2, CHFR, PKIA, MYC, BRCA1, TGFB2*
Regulation of cyclase activity	3.5	6	0.027	*EDNRB, GRM3, ADCY7, GALR1, GRM6, GNAS*
Regulation of camp biosynthetic process	3.5	6	0.029	*EDNRB, GRM3, ADCY7, GALR1, GRM6, GNAS*
Regulation of lyase activity	3.5	6	0.029	*EDNRB, GRM3, ADCY7, GALR1, GRM6, GNAS*
Regulation of camp metabolic process	3.4	6	0.032	*EDNRB, GRM3, ADCY7, GALR1, GRM6, GNAS*

Positive regulation of apoptosis	1.9	14	0.033	*DCC, CD3E, SKP2, TRIO, YWHAE, BRCA1, TGFB2, JMY, TNFRSF19, IL12B, MYC, PDCD7, DNM2, FGD4*
Hepatocyte growth factor receptor signaling pathway	58.3	2	0.034	*MET, HGF*
Positive regulation of programmed cell death	1.9	14	0.035	*DCC, CD3E, SKP2, TRIO, YWHAE, BRCA1, TGFB2, JMY, TNFRSF19, IL12B, MYC, PDCD7, DNM2, FGD4*
Positive regulation of cell death	1.9	14	0.036	*DCC, CD3E, SKP2, TRIO, YWHAE, BRCA1, TGFB2, JMY, TNFRSF19, IL12B, MYC, PDCD7, DNM2, FGD4*
Lymphocyte activation during immune response	9.7	3	0.037	*RELB, IL12B, ADA*
Cell death	1.6	20	0.039	*E2F1, MEF2C, DCC, TAOK2, FGF14, TRIO, YWHAE, BRCA1, RRAGC, TGFB2, ATXN2, BNIPL, RASSF5, APOL1, ATG5, TNFRSF19, TIAF1, MYC, PDCD7, FGD4*
Regulation of nucleotide biosynthetic process	3.2	6	0.04	*EDNRB, GRM3, ADCY7, GALR1, GRM6, GNAS*
Regulation of cyclic nucleotide biosynthetic process	3.2	6	0.04	*EDNRB, GRM3, ADCY7, GALR1, GRM6, GNAS*
Death	1.6	20	0.042	*E2F1, MEF2C, DCC, TAOK2, FGF14, TRIO, YWHAE, BRCA1, RRAGC, TGFB2, ATXN2, BNIPL, RASSF5, APOL1, ATG5, TNFRSF19, TIAF1, MYC, PDCD7, FGD4*
Regulation of cyclic nucleotide metabolic process	3.1	6	0.044	*EDNRB, GRM3, ADCY7, GALR1, GRM6, GNAS*
Cell migration	2.1	10	0.047	*DCC, PHOX2B, EDNRB, TAOK2, MET, ENPEP, IL12B, SRF, YWHAE, TGFB2*
Second-messenger-mediated signaling	2.2	9	0.048	*EDNRB, P2RY6, HRH1, ADCY7, GALR1, GRM6, HIST1H4E, GNAS, FEN1*
Regulation of nucleotide metabolic process	3	6	0.049	*EDNRB, GRM3, ADCY7, GALR1, GRM6, GNAS*
Regulation of survival gene product expression	8.3	3	0.049	*MEF2C, SKP2, MYC*

Table S7 . Gene ontology enrichment analysis using the genes with DMCs in LT

GO term	Fold Enrichment	Count	*p* value	Genes
Response to wounding	2.9	13	0.002	*C3AR1, KLK8, S100A8, NDST1, IL27, F7, SYT7, EPHA3, FGG, HNF4A, F2, MS4A2, SERPIND1*
Regulation of cell growth	4.2	7	0.006	*HNF4A, SEMA3F, RB1, ESM1, GREM1, CDH4, ALOX12*
Regulation of body fluid levels	5	6	0.007	*FGG, HNF4A, OXT, F2, SERPIND1, F7*
Regulation of cell size	4	7	0.008	*HNF4A, SEMA3F, TGFBR3, RB1, GREM1, CDH4, ALOX12*
Regulation of cellular component size	3.4	8	0.008	*HNF4A, SEMA3F, TGFBR3, RB1, EPB49, GREM1, CDH4, ALOX12*
Blood coagulation	5.7	5	0.011	*FGG, HNF4A, F2, SERPIND1, F7*
Coagulation	5.7	5	0.011	*FGG, HNF4A, F2, SERPIND1, F7*
Homeostatic process	2.2	14	0.011	*SRI, C3AR1, OXT, CCDC47, RB1, EPHA3, HNF4A, APOC4, PLN, F2, NPC1L1, TGFBR3, DYRK3, MT1H*
Hemostasis	5.4	5	0.013	*FGG, HNF4A, F2, SERPIND1, F7*
Regulation of homeostatic process	5.1	5	0.016	*KLK8, OXT, F2, SPI1, MS4A2*
Di-, tri-valent inorganic cation homeostasis	3.4	7	0.016	*SRI, C3AR1, OXT, PLN, F2, CCDC47, MT1H*
Superoxide metabolic process	14	3	0.019	*NOXO1, NOXA1, ALOX12*
Wound healing	3.7	6	0.023	*FGG, HNF4A, F2, SERPIND1, F7, SYT7*
Regulation of nervous system development	3.6	6	0.024	*HOXA2, CDK5R1, KLK8, SEMA3F, OXT, CDH4*
Regulation of cell morphogenesis	4.5	5	0.025	*KLK8, SEMA3F, TGFBR3, FBLIM1, CDH4*
Regulation of neuron differentiation	4.4	5	0.027	*HOXA2, CDK5R1, KLK8, SEMA3F, CDH4*
Regulation of cell morphogenesis involved in differentiation	6.1	4	0.028	*KLK8, SEMA3F, TGFBR3, CDH4*
Chemical homeostasis	2.3	10	0.030	*SRI, C3AR1, HNF4A, APOC4,*

				OXT, PLN, F2, NPC1L1, CCDC47, MT1H
Regulation of cell development	3.4	6	0.031	*HOXA2, CDK5R1, KLK8, SEMA3F, TGFBR3, CDH4*
Negative regulation of cellular component organization	4.1	5	0.033	*IRAK3, KLK8, SEMA3F, TGFBR3, EPB49*
Calcium ion transport	4.1	5	0.033	*SRI, CATSPER2, PLN, F2, PSEN2*
Response to prostaglandin stimulus	58.3	2	0.034	*OXT, TGFBR3*
Response to prostaglandin E stimulus	58.3	2	0.034	*OXT, TGFBR3*
Muscle organ development	3.3	6	0.034	*SRI, MSX1, MAPK12, PLN, VAMP5, TGFBR3*
Cation homeostasis	2.9	7	0.035	*SRI, C3AR1, OXT, PLN, F2, CCDC47, MT1H*
Negative regulation of cell growth	5.1	4	0.044	*HNF4A, SEMA3F, RB1, GREM1*
Cellular di-, tri-valent inorganic cation homeostasis	3.1	6	0.044	*SRI, C3AR1, OXT, PLN, F2, MT1H*
Myeloid cell differentiation	5	4	0.045	*PSEN2, TGFBR3, DYRK3, RB1*

Table S8. Differential gene expression in WB in the comparison between DO and NDO groups

Symbol	Accession	logFC	*p* value
RAP1GAP	NM_002885.1	1.430	0.001
UTS2	NM_006786.2	1.307	0.009
AMFR	NM_001144.4	1.245	0.020
NFIX	NM_002501.2	1.175	0.023
LOC100131164	XM_001721919.1	1.160	0.002
MCOLN1	NM_020533.1	1.152	<0.001
C16ORF35	NM_001039476.1	1.126	<0.001
RNF182	NM_152737.2	1.101	0.001
SLC4A1	NM_000342.2	1.096	<0.001
HSPC157	NR_023918.1	1.068	0.037
SOCS1	NM_003745.1	1.064	0.008
KRT1	NM_006121.3	1.004	0.001
USF1	NM_007122.3	0.993	0.013

UTS2	NM_021995.1	0.969	0.043
C17ORF97	NM_001013672.3	0.948	0.045
ZDHHC19	NM_001039617.1	0.912	0.001
C16ORF35	NM_012075.1	0.899	<0.001
RBM38	NM_017495.4	0.880	0.029
CMBL	NM_138809.3	0.853	0.032
VWCE	NM_152718.2	0.841	0.005
LOC100128714	XM_001722756.1	0.833	<0.001
TSTA3	NM_003313.2	0.828	0.001
NR1D1	NM_021724.2	0.827	0.002
TMEM86B	NM_173804.3	0.827	0.005
ACSL6	NM_001009185.1	0.823	<0.001
TRIM58	NM_015431.3	0.821	0.002
SCARNA17	NR_003003.2	0.820	<0.001
PLEK2	NM_016445.1	0.813	0.007
SLC6A10P	NR_003083.2	0.810	0.010
SLC22A16	NM_033125.2	-0.843	0.001
NAPSA	NM_004851.1	-0.849	0.020
LACTB2	NM_016027.1	-0.870	0.001
LOC653071	XM_930721.1	-0.885	0.006
ODF2L	NM_001007022.1	-0.893	0.003
SPAST	NM_199436.1	-0.935	0.000
ORM2	NM_000608.2	-0.975	<0.001
COPG2	NM_012133.2	-0.977	0.005
NKX3-1	NM_006167.2	-0.988	0.002
LOC283547	XM_378454.3	-1.007	<0.001
PLCB1	NM_015192.2	-1.013	0.004
ORM1	NM_000607.1	-1.067	0.050
MFF	NM_020194.4	-1.273	0.040
LOC253039	NR_024408.1	-1.348	0.002

Table S9. Differential gene expression in SAT in the comparison between DO and NDO groups

Symbol	Accession	logFC	*p* value
CCL20	NM_004591.1	1.310	0.032
SLAMF1	NM_003037.1	1.068	0.034
AMFR	NM_001144.4	0.955	0.005
PTX3	NM_002852.2	0.937	0.035
ACTA1	NM_001100.3	0.862	0.013
LIPG	NM_006033.2	0.831	0.021
TNC	NM_002160.2	0.828	0.011
EDN1	NM_001955.2	0.815	0.018
SERPINA3	NM_001085.4	0.809	0.026
LOC645313	XR_017585.2	-0.829	0.026
MS4A4A	NM_148975.1	-0.829	0.003
ITGB1BP1	NM_022334.3	-0.854	0.020
MIR1974	NR_031738.1	-0.966	0.011
SEL1L2	NM_025229.1	-1.032	0.004
STMN2	NM_007029.2	-1.127	<0.001
LOC644936	NR_004845.1	-1.164	0.016
STMN2	NM_007029.2	-1.333	<0.001

Table S10. Differential gene expression in VAT in the comparison between DO and NDO groups

Symbol	Accession	logFC	*p* value
AMFR	NM_001144.4	1.149	0.034
G3BP2	NM_203504.1	1.020	0.021
G3BP2	NM_203504.1	0.829	0.022
GZMH	NM_033423.3	-0.832	<0.001
RNU1A3	NR_004430.1	-0.860	0.028
DHRS9	NM_005771.3	-0.860	0.036
LOC648984	XM_938063.1	-0.868	<0.001
CCL18	NM_002988.2	-0.884	0.037
NR4A3	NM_173199.1	-0.996	0.006
ACP5	NM_001611.2	-1.011	0.011
RNU1-3	NR_004408.1	-1.015	0.046
ALAS2	NM_001037968.1	-1.047	0.009
HBE1	NM_005330.3	-1.070	0.004
RNU1G2	NR_004426.1	-1.081	0.038
RNU1-5	NR_004400.1	-1.088	0.028
HSPA1B	NM_005346.3	-1.100	0.034
DEFB1	NM_005218.3	-1.133	0.012
KLF4	NM_004235.3	-1.225	0.038
KLF4	NM_004235.3	-1.366	0.044
JUN	NM_002228.3	-1.389	0.031
LOC644936	NR_004845.1	-1.402	0.037
HBD	NM_000519.3	-1.443	0.001
NR4A2	NM_006186.2	-1.547	0.034
HBA1	NM_000558.3	-1.731	0.006
LOC731682	XM_001129369.1	-1.877	0.010
HBG2	NM_000184.2	-1.899	0.001
HBG1	NM_000559.2	-2.020	0.001
FOS	NM_005252.2	-2.757	0.021
FOSB	NM_006732.1	-3.175	0.024

Table S11. Differential gene expression in LT in the comparison between DO and NDO groups

Symbol	Accession	logFC	*p* value
HLA-DRB1	NM_002124.1	1.447	0.043
PLA2G2A	NM_000300.2	1.179	0.026
LOC729708	XM_001725700.1	-1.053	0.039
LOC100132564	XM_001713808.1	-1.091	0.049

Table S12. List of genes with correlation between alteration of DNA methylation and differential gene expression in WB

Symbol	Target ID	Delta Beta	*p* value (Delta Beta)	log FC	*p* value (logFC)
BRDT	cg14732540	0.086	0.008	-0.132	0.007
PSG6	cg11151665	0.064	<0.001	-0.125	0.029
PPP2R2D	cg21750602	0.039	0.044	-0.196	0.029
GPR175	cg13728650	0.031	0.006	-0.193	0.037
KRT13	cg10742225	0.027	0.017	-0.132	0.015
C21orf94	cg17266238	0.025	0.032	-0.140	0.047
PSG6	cg17642353	0.022	0.040	-0.125	0.029
HBQ1	cg17714030	0.019	0.008	-0.570	0.001
HBD	cg20609368	0.016	0.041	-1.443	0.001
SLC7A6OS	cg27077685	0.010	0.025	-0.283	0.024
STEAP3	cg04749104	0.010	0.038	-0.176	0.010
THSD1	cg16114640	0.009	0.013	-0.169	0.004
PRMT2	cg06725035	0.008	0.018	-0.295	0.007
ASAH1	cg13563405	-0.004	0.048	0.164	0.004
TTC9C	cg24515202	-0.004	0.027	0.195	0.049
APBA1	cg15840658	-0.005	0.037	0.138	0.037
APBA1	cg15623573	-0.005	0.048	0.138	0.037
BCKDHB	cg15256539	-0.006	0.020	0.191	0.048
BCKDHB	cg15256539	-0.006	0.020	0.284	0.009
PPM1J	cg12687922	-0.006	0.024	0.092	0.039
RET	cg05621401	-0.008	0.014	0.155	0.035
LARGE	cg05670348	-0.008	0.028	0.301	0.019
MEIS2	cg02917381	-0.008	0.005	0.109	0.027
RBKS	cg06869899	-0.009	0.017	0.176	0.036
MBD6	cg25107978	-0.012	0.015	0.334	0.043

ERCC8	cg11649654	-0.012	0.047	0.239	0.001
PPP1R3D	cg08966023	-0.013	0.044	0.165	0.045
GOSR1	cg19098314	-0.014	0.002	0.139	0.033
RFK	cg13789236	-0.014	0.009	0.127	0.032
PLEKHA1	cg01246254	-0.020	<0.001	0.282	0.040
IRX3	cg24662961	-0.020	0.003	0.774	0.021
DPH2	cg25955969	-0.026	0.035	0.163	0.026
IGF2	cg02166532	-0.027	0.008	0.132	0.024
LRPPRC	cg26538116	-0.027	0.013	0.227	0.024
SNX16	cg06027949	-0.034	0.011	0.435	0.032
FLJ44060	cg08489623	-0.041	0.032	0.128	0.034
CRB3	cg03258472	-0.046	0.026	0.173	0.033
BARHL2	cg17241310	-0.046	0.036	0.255	0.004
C14orf105	cg19903229	-0.051	0.013	0.132	0.012
SNX4	cg16468910	-0.054	0.022	0.212	0.015
HMP19	cg07884019	-0.062	0.047	0.119	0.031
EDNRB	cg24745738	-0.101	0.001	0.193	0.026

Table S13. List of genes with correlation between alteration of DNA methylation and differential gene expression in SAT

Symbol	Target ID	Delta Beta	*p* value (Delta Beta)	log FC	*p* value (logFC)
LEP	cg12782180	0.056	0.008	-0.352	0.022
ZNF3	cg00929606	0.054	0.023	-0.142	0.001
CAPSL	cg24202119	0.028	0.040	-0.166	0.006
KCNQ1	cg17229197	0.026	0.019	-0.293	<0.001
KCNQ1	cg27491887	0.024	0.037	-0.293	<0.001
C1QC	cg11393848	0.023	0.029	-0.600	0.007
CTSE	cg04457794	0.015	0.021	-0.232	0.018
CLTA	cg26966384	0.015	0.035	-0.206	0.003
CLTA	cg26966384	0.015	0.035	-0.174	0.027
NIT1	cg10053203	0.015	0.010	-0.284	0.006
ST7L	cg01183669	0.015	0.009	-0.145	0.031
OPRM1	cg14262937	0.014	0.022	-0.129	0.013
OPRM1	cg14262937	0.014	0.022	-0.198	<0.001
PTGER3	cg12739034	0.012	0.009	-0.635	0.001
DHFR	cg16161425	0.011	0.040	-0.170	0.047

PTPRO	cg27196745	0.008	0.043	-0.447	0.011
POFUT1	cg25811820	0.006	0.016	-0.078	0.048
ULBP1	cg25589890	0.006	0.041	-0.173	0.015
SFRS3	cg08027745	0.005	0.044	-0.406	0.017
TSC22D4	cg08487374	0.005	0.016	-0.292	0.010
CCDC5	cg18307767	0.004	0.046	-0.219	0.047
POP5	cg07236358	0.004	0.042	-0.310	0.031
F5	cg09891761	-0.004	0.032	0.559	0.007
DICER1	cg16184930	-0.005	0.016	0.146	0.011
GUCY1A2	cg23984434	-0.005	0.030	0.131	0.021
USP48	cg20956373	-0.005	0.028	0.347	0.048
MLL5	cg05566397	-0.006	0.045	0.542	0.008
AMPH	cg10293925	-0.007	0.002	0.660	0.011
YWHAB	cg07064406	-0.008	0.047	0.156	0.045
ZNF273	cg05306735	-0.013	0.016	0.189	0.032
CUGBP2	cg15777781	-0.015	0.014	0.338	0.014
LRRC39	cg26117431	-0.015	0.009	0.129	0.005
TUB	cg15480475	-0.015	0.019	0.427	0.041
TOMM34	cg11454415	-0.017	0.014	0.325	0.017
DNM1L	cg27387222	-0.017	0.017	0.327	0.025
CLN8	cg23833896	-0.020	0.006	0.227	0.023
C1orf124	cg15459773	-0.021	0.046	0.183	0.005
C1orf124	cg15459773	-0.021	0.046	0.233	0.031
C11orf47	cg18103150	-0.021	0.012	0.211	0.031
PDE4D	cg05992340	-0.022	0.008	0.405	0.033
SCT	cg05782292	-0.025	0.048	0.075	0.038
TCAP	cg01680823	-0.026	0.049	0.228	0.011
ADRA1D	cg09614401	-0.030	0.039	0.102	0.017
BVES	cg20624391	-0.034	0.023	0.073	0.045
NR2E3	cg18860847	-0.034	0.038	0.098	0.016
SYNJ2	cg13645811	-0.034	0.001	0.553	0.002
RIMS3	cg08394377	-0.036	0.016	0.291	0.030
RUNX2	cg05996042	-0.037	0.003	0.123	0.042
ADPRHL1	cg00463202	-0.045	0.010	0.091	0.007
P4HA3	cg25599211	-0.048	0.015	0.085	0.025
DST	cg04452713	-0.051	0.003	0.319	0.024
MGAT4C	cg18344063	-0.062	0.047	0.121	0.038

Table S14. List of genes with correlation between alteration of DNA methylation and differential gene expression in VAT

Symbol	Target ID	Delta Beta	*p* value (Delta Beta)	log FC	*p* value (logFC)
BRDT	cg14732540	0.086	0.008	-0.132	0.007
PSG6	cg11151665	0.064	<0.001	-0.125	0.029
PPP2R2D	cg21750602	0.039	0.044	-0.196	0.029
GPR175	cg13728650	0.031	0.006	-0.193	0.037
KRT13	cg10742225	0.027	0.017	-0.132	0.015
C21orf94	cg17266238	0.025	0.032	-0.140	0.047
PSG6	cg17642353	0.022	0.040	-0.125	0.029
HBQ1	cg17714030	0.019	0.008	-0.570	0.001
HBD	cg20609368	0.016	0.041	-1.443	0.001
SLC7A6OS	cg27077685	0.010	0.025	-0.283	0.024
STEAP3	cg04749104	0.010	0.038	-0.176	0.010
THSD1	cg16114640	0.009	0.013	-0.169	0.004
PRMT2	cg06725035	0.008	0.018	-0.295	0.007
ASAH1	cg13563405	-0.004	0.048	0.164	0.004
TTC9C	cg24515202	-0.004	0.027	0.195	0.049
APBA1	cg15840658	-0.005	0.037	0.138	0.037
APBA1	cg15623573	-0.005	0.048	0.138	0.037
BCKDHB	cg15256539	-0.006	0.020	0.191	0.048
BCKDHB	cg15256539	-0.006	0.020	0.284	0.009
PPM1J	cg12687922	-0.006	0.024	0.092	0.039
RET	cg05621401	-0.008	0.014	0.155	0.035
LARGE	cg05670348	-0.008	0.028	0.301	0.019
MEIS2	cg02917381	-0.008	0.005	0.109	0.027
RBKS	cg06869899	-0.009	0.017	0.176	0.036
MBD6	cg25107978	-0.012	0.015	0.334	0.043
ERCC8	cg11649654	-0.012	0.047	0.239	0.001
PPP1R3D	cg08966023	-0.013	0.044	0.165	0.045
GOSR1	cg19098314	-0.014	0.002	0.139	0.033
RFK	cg13789236	-0.014	0.009	0.127	0.032
PLEKHA1	cg01246254	-0.020	<0.001	0.282	0.040
IRX3	cg24662961	-0.020	0.003	0.774	0.021
DPH2	cg25955969	-0.026	0.035	0.163	0.026
IGF2	cg02166532	-0.027	0.008	0.132	0.024
LRPPRC	cg26538116	-0.027	0.013	0.227	0.024
SNX16	cg06027949	-0.034	0.011	0.435	0.032

FLJ44060	cg08489623	-0.041	0.032	0.128	0.034
CRB3	cg03258472	-0.046	0.026	0.173	0.033
BARHL2	cg17241310	-0.046	0.036	0.255	0.004
C14orf105	cg19903229	-0.051	0.013	0.132	0.012
SNX4	cg16468910	-0.054	0.022	0.212	0.015
HMP19	cg07884019	-0.062	0.047	0.119	0.031
EDNRB	cg24745738	-0.101	0.001	0.193	0.026

Table S15. List of genes with correlation between alteration of DNA methylation and differential gene expression in LT

Symbol	Target ID	Delta Beta	*p* value (Delta Beta)	log FC	*p* value (logFC)
SLC12A8	cg14391622	0.049	0.021	-0.353	0.034
CACNB4	cg18272264	0.042	0.011	-0.152	0.033
FAM3B	cg03158400	0.040	0.040	-0.128	0.037
SYK	cg10025443	0.038	0.022	-0.133	0.047
CTBS	cg08380539	0.004	0.046	-0.263	0.020
MARCH8	cg02702510	-0.011	0.027	0.070	0.041
SON	cg26265060	-0.027	0.050	0.064	0.050
KCNJ1	cg14481339	-0.033	0.022	0.096	0.034
TMEM92	cg25949363	-0.035	0.038	0.116	0.031
PLA2G2A	cg11037787	-0.042	0.025	1.179	0.026
NRG4	cg13044277	-0.043	0.028	0.236	0.036
PNLIPRP1	cg27600794	-0.044	0.021	0.110	0.014
SYT7	cg02104644	-0.051	0.046	0.138	0.033
LTBR	cg15784615	-0.052	0.013	0.250	0.028
CATSPER2	cg23685580	-0.052	0.027	0.078	0.032
LPAL2	cg15398520	-0.058	0.027	0.107	0.010
NCALD	cg01484156	-0.059	<0.001	0.266	0.013
ZDHHC11	cg20584011	-0.059	0.002	0.763	0.007
LGTN	cg16639185	-0.060	0.031	0.241	0.031
ZDHHC11	cg18429742	-0.078	0.016	0.763	0.007
OXT	cg26955850	-0.080	0.050	0.802	0.037
PRSS21	cg22730830	-0.113	0.003	0.132	0.012
OXT	cg26267561	-0.154	0.001	0.802	0.037

Table S16. Gene ontology enrichment analysis using the genes with correlation between alteration of DNA methylation and differential gene expression

Tissue	Term	Count	*p* value	Genes
**WB**	GO:0042981~regulation of apoptosis	9	0.011	*MSH6, CASP9, SNCB, DEDD, PLEKHG5, HSPA5, IGFBP3, CLN8, GCH1*
	GO:0043067~regulation of programmed cell death	9	0.012	*MSH6, CASP9, SNCB, DEDD, PLEKHG5, HSPA5, IGFBP3, CLN8, GCH1*
	GO:0010941~regulation of cell death	9	0.012	*MSH6, CASP9, SNCB, DEDD, PLEKHG5, HSPA5, IGFBP3, CLN8, GCH1*
	GO:0009416~response to light stimulus	4	0.016	*MSH6, CASP9, NR2E3, SCARA3*
	GO:0009411~response to UV	3	0.022	*MSH6, CASP9, SCARA3*
	GO:0043065~positive regulation of apoptosis	6	0.024	*MSH6, CASP9, DEDD, PLEKHG5, IGFBP3, GCH1*
	GO:0043068~positive regulation of programmed cell death	6	0.025	*MSH6, CASP9, DEDD, PLEKHG5, IGFBP3, GCH1*
	GO:0006979~response to oxidative stress	4	0.025	*UCP2, TXNRD2, SCARA3, CLN8*
	GO:0010942~positive regulation of cell death	6	0.025	*MSH6, CASP9, DEDD, PLEKHG5, IGFBP3, GCH1*
	GO:0050884~neuromuscular process controlling posture	2	0.034	*CLN8, GCH1*
	GO:0045773~positive regulation of axon extension	2	0.034	*LIMK1, MAPT*
	GO:0009314~response to radiation	4	0.041	*MSH6, CASP9, NR2E3, SCARA3*
	GO:0000305~response to oxygen radical	2	0.041	*UCP2, TXNRD2*
**SAT**	GO:0007005~mitochondrion organization	4	0.007	*DNM1L, SYNJ2, CLN8, TOMM34*
	GO:0008015~blood circulation	4	0.015	*F5, TCAP, KCNQ1, ADRA1D*
	GO:0003013~circulatory system process	4	0.015	*F5, TCAP, KCNQ1, ADRA1D*
	GO:0040029~regulation of gene expression, epigenetic	3	0.020	*MLL5, DICER1, KCNQ1*
	GO:0007188~G-protein signaling, coupled to cAMP nucleotide second messenger	3	0.024	*OPRM1, PTGER3, ADRA1D*
	GO:0019933~cAMP-mediated signaling	3	0.029	*OPRM1, PTGER3, ADRA1D*

	GO:0007187~G-protein signaling, coupled to cyclic nucleotide second messenger	3	0.041	*OPRM1, PTGER3, ADRA1D*
**VAT**	GO:0007497~posterior midgut development	2	0.004	*EDNRB, RET*
	GO:0048484~enteric nervous system development	2	0.020	*EDNRB, RET*
	GO:0015671~oxygen transport	2	0.027	*HBQ1, HBD*
**LT**	GO:0046903~secretion	5	0.000	*OXT, FAM3B, CACNB4, SYK, KCNJ1*
	GO:0003001~generation of a signal involved in cell-cell signaling	3	0.004	*FAM3B, CACNB4, SYK*
	GO:0030001~metal ion transport	4	0.016	*SLC12A8, CATSPER2, CACNB4, KCNJ1*
	GO:0006812~cation transport	4	0.026	*SLC12A8, CATSPER2, CACNB4, KCNJ1*
	GO:0048584~positive regulation of response to stimulus	3	0.031	*PLA2G2A, CACNB4, SYK*
	GO:0007267~cell-cell signaling	4	0.032	*OXT, FAM3B, CACNB4, SYK*
	GO:0050851~antigen receptor-mediated signaling pathway	2	0.038	*CACNB4, SYK*
	GO:0051094~positive regulation of developmental process	3	0.042	*OXT, PLA2G2A, SYK*
	GO:0002429~immune response-activating cell surface receptor signaling pathway	2	0.045	*CACNB4, SYK*
